# Amino-7,8-dihydro-4H-chromenone derivatives as potential inhibitors of acetylcholinesterase and butyrylcholinesterase for Alzheimer’s disease management; in vitro and in silico study

**DOI:** 10.1186/s13065-024-01170-x

**Published:** 2024-04-10

**Authors:** Ali Asadipour, Yaghoub Pourshojaei, Moein Mansouri, Elham Mahdavizadeh, Cambyz Irajie, Javad Mottaghipisheh, Ehsan Faghih-Mirzaei, Mohammad Mahdavi, Aida Iraji

**Affiliations:** 1 Department of Medicinal Chemistry, Faculty of Pharmacy, Kerman University of Medical Sciences, Kerman, Iran; 2 Extremophile and Productive Microorganisms Research Center, Kerman University of Medical Sciences, Kerman, Iran; 3 Department of Medical Biotechnology, School of Advanced Medical Sciences and Technologies, Shiraz University of Medical Sciences, Shiraz, Iran; 4 Department of Aquatic Sciences and Assessment, Swedish University of Agricultural Sciences, 75007 Uppsala, Sweden; 5 Research Center for Traditional Medicine and History of Medicine, Department of Persian Medicine, School of Medicine, Shiraz University of Medical Sciences, Shiraz, Iran; 6 Endocrinology and Metabolism Research Center, Endocrinology and Metabolism Clinical Sciences Institute, Tehran University of Medical Sciences, Tehran, Iran; 7 Stem Cells Technology Research Center, Shiraz University of Medical Sciences, Shiraz, Iran

**Keywords:** Acetylcholinesterase, Alzheimer’s disease, Butyrylcholinesterase, Chromenone, Kinetic, Molecular dynamics

## Abstract

In this article, we present the design and synthesis of amino-7,8-dihydro-4H-chromenone derivatives as possible inhibitors of acetylcholinesterase (AChE) and butyrylcholinesterase (BChE) for the management of Alzheimer’s disease (AD). The target compounds were evaluated against AChE and BChE in vitro, and **4k** exhibited good potency against BChE (IC_50_ = 0.65 ± 0.13 µM) compared with donepezil used as a positive control. Kinetic studies revealed that compound **4k** exhibited a competitive-type inhibition with a *K*_*i*_ value of 0.55 µM. Molecular docking and molecular dynamics simulations further supported the rationality of our design strategy, as **4k** showed promising binding interactions with the active sites of BChE. Overall, our findings highlight the potential of amino-7,8-dihydro-4H-chromenone derivatives as promising candidates for developing novel therapeutics targeting cholinesterase in managing AD.

**Supplementary Information:**

The online version contains supplementary material available at 10.1186/s13065-024-01170-x.

## Introduction

Alzheimer’s disease (AD) represents a significant public health challenge, especially as the most common form of dementia affecting the elderly. The global prevalence of AD is a growing concern, with approximately 50 million individuals diagnosed with dementia in 2018, and this number is projected to rise dramatically to an alarming 132 million by 2060. This escalating burden poses immense emotional and financial challenges for individuals and society [1].

Despite extensive research, the exact etiology of AD remains unknown. However, various factors are thought to contribute to its pathogenesis, including amyloid β (Aβ) deposits, τ-protein aggregation, oxidative stress, and the depletion of acetylcholine (ACh) levels in critical brain regions such as the hippocampus and cortex [2–4]. The cholinergic hypothesis suggests that reducing ACh, a neurotransmitter crucial for memory and learning, maybe a potential cause of AD. The loss and dysfunction of cholinergic transmission, accompanied by reduced acetylcholine neurotransmitters, are major molecular hallmarks of AD. Acetylcholinesterase (AChE) plays a pivotal role in the hydrolysis of acetylcholine, leading to the breakdown of this essential neurotransmitter in the synaptic cleft. Conversely, in the later stages of AD, there is an observed increase in butyrylcholinesterase (BChE), which may serve as a compensatory mechanism to counterbalance the reduced AChE activity [2, 5].

From a pharmacological standpoint, the current approach to AD management primarily relies on cholinesterase (ChE) inhibitors such as donepezil, rivastigmine, and galantamine. These drugs elevate ChE levels, enhancing cholinergic neurotransmission and mitigating cognitive decline [6]. On the other hand, memantine, with its unique mechanism of action as an N-methyl-D-aspartate (NMDA) receptor antagonist, aims to modulate excessive NMDA receptor activation, providing an alternative therapeutic strategy [7].

**Fig. 1:**
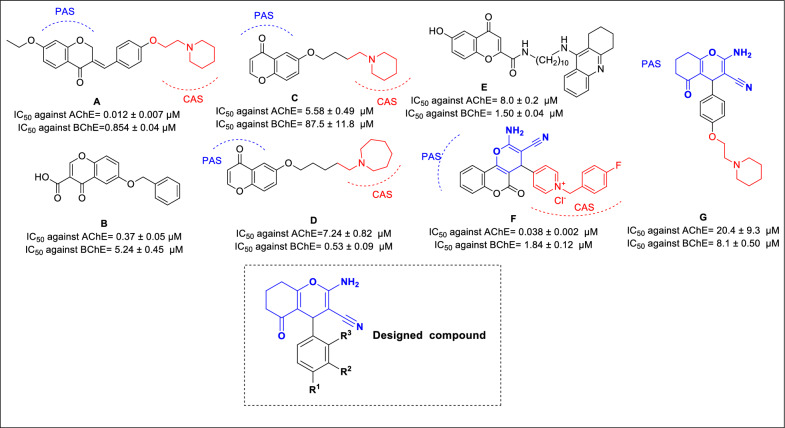
The previously reported chromenone derivatives as ChE inhibitors and newly designed compound

Chromenone, characterized by its heterocyclic ring, holds a prominent position in medicinal chemistry as a versatile building block for synthesizing various pharmacologically active agents. Its prevalence is not limited to natural compounds but has also been the subject of extensive synthetic entry, exploring its potential biological activities in drug discovery [8]. Furthermore, chromone derivatives have demonstrated compelling antioxidant, anti-inflammatory, and anti-AD potentials [9]. In 2015, benzylidene chroman-4-one derivatives were discovered, demonstrating potent activity against AChE with IC_50_ values ranging from 0.122 μM to 0.207 μM. The structure–activity relationships (SARs) indicated that the most favorable potency was achieved with a cyclic amine and ethoxy (n = 1) substituent (compound **A,** Fig. 1). Docking studies revealed that the Chromenone ring of these compounds was oriented towards the peripheral anionic site (PAS), while the piperine moiety occupied the catalytic anionic site (CAS) pocket of AChE [10]. Another successful approach involved combining chromanone with a benzyl tail of donepezil (compound **B,** Fig. 1). The most potent analog demonstrated promising inhibitory behavior against both AChE (IC_50_ = 0.37 μM) and BChE (IC_50_ = 5.24 μM), along with good blood–brain barrier (BBB) permeability (5.4 ± 0.3 × 10^−6^ cm s^−1^) [11]. In addition, Michael Gütschow et al. systematically explored a library of chromen-4-ones with different structural features. Compound **C** exhibited notable potency against AChE, while compound **D** displayed increased potency against BChE due to an increase in bulkiness favored BChE inhibition [12]. Furthermore, tacrine − 4-Oxo-4H-chromene hybrids (**E,** Fig. 1) were identified as potential cholinesterase inhibitors, demonstrating good BBB permeability with a value of 23.1 ± 0.1 × 10^−6^ cm s^−1^) [13].

Previous studies have highlighted the essential roles of the amine and nitrile groups on the chromenone ring in cholinesterase inhibition. The amine group engages in hydrogen bonding interactions with the PAS pocket, and the nitrile moiety occupies an optimum position for interactions with the PAS pocket binding site (compounds **F** and **G,** Fig. 1). Understanding these interactions helps researchers design and modify chromenone-based compounds for more effective cholinesterase inhibition [14, 15].

In this study, we designed and synthesized a series of amino-7,8-dihydro-4H-chromenone compounds as potential inhibitors of ChE. The synthesis of these compounds involved a tandem Knoevenagel-Michael reaction approach to explore various substitutions on the phenyl ring attached to the chromen-4-one scaffold. To assess their anti-ChE activity, we employed a modified Ellman’s method. Among the synthesized derivatives, the most promising compound was selected for a kinetic study to understand its interactions with the respective enzyme. Furthermore, in silico assessments, including molecular docking and molecular dynamic studies, were conducted to gain valuable insights into the compounds’ interactions with the enzymes. These computational analyses provided a deeper understanding of the binding modes and interactions at the molecular level, which can contribute to the rational design and optimization of potential ChE inhibitors for therapeutic applications.

## Results and discussion

### Synthesis

**Scheme 1:**
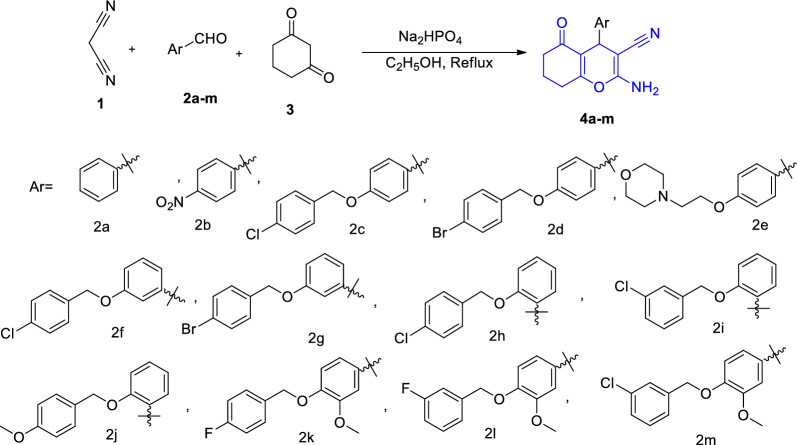
Synthetic route to the amino-7,8-dihydro-4H-chromenone derivatives **4a–m**

The target compounds were prepared with good yield in the 70–90% range, and the optimum yield of products was achieved after refluxing the reactants in the 4–6 h range. For the synthesis of 4H-chromene derivatives **4a–m**, firstly, malononitrile **1**, substituted benzaldehyde **2a–m** and sodium dihydrogen phosphate as a safe and efficient catalyst were reacted in ethanol to produce the corresponding benzylidenemalononitrile compounds. The mixture was stirred for 2 to 4 h. Then, 1,3-cyclohexanedione and additional sodium dihydrogen phosphate were added to the system, and the mixture was kept under reflux conditions to obtain the final products during a Michael reaction and cyclization. The completion of the reaction was checked by thin-layer chromatography. After that, water was added to the latter mixture, and products were filtered off and recrystallized in ethanol to give pure products **4a–m**. The structures of the new products were confirmed using FT-IR, ^1^H-NMR, ^13^C-NMR spectroscopy, elemental analysis (C, H, N), and for known products by comparing their melting points with the reported ones (Scheme 1 and Table 1).

**Table 1:** The results of the synthesis of different amino-7,8-dihydro-4H-chromenone derivatives

Entry	Product	Time (h)	Yield (%)^a^	m.p^b^. (lit)^c^(°C)
1		4	90	235–236 (232–234)^c^ [16]
2		5	88	214–215 (213–214)^c^ [16]
3		5	90	183–185^b^
4		4.5	87	214–216^b^
5		4	90	188–190^c^
6		5	86	224–226^b^
7		6	85	191–194^b^
8		5	90	201–204^b^
9		6	90	224–226^b^
10		5	85	204–207^b^
11		6	75	215–218^b^
12		6	90	227–230^b^
13		4	70	228–230^b^

^a^Isolated yield, ^b^novel product, ^c^known product

### Evaluation of AChE inhibition

The target compounds **4a–m** were evaluated for their in vitro inhibitory activities against AChE and BChE compared with donepezil as a standard drug (Table 2).

**Table 2:** The anti-AChE and anti-BChE activity of **4a-m** derivatives^[a]^


Entry	R^1^	R^2^	R^3^	ACHE % Inhibition at 50 µM	BChE % Inhibition at 50 µM	BChE (IC_50_, µM)
**4a**	H	H	H	21.02 ± 1.31	39.77 ± 1.39^†^	–
**4b**	–NO_2_	H	H	28.89 ± 2.56*	58.72 ± 1.18^†^	27.42 ± 2.01
**4c**		H	H	23.57 ± 1.20	77.67 ± 3.77^†^	0.89 ± 0.24
**4d**		H	H	23.42 ± 1.00	67.82 ± 1.70^†^	1.19 ± 0.31
**4e**		H	H	34.44 ± 3.10*	58.94 ± 0.93^†^	26.35 ± 3.34
**4f**	H		H	4.42 ± 0.57*	61.96 ± 7.24^†^	5.70 ± 0.68
**4g**	H		H	14.97 ± 2.59*	57.29 ± 9.75^†^	13.06 ± 2.59
**4h**	H	H		7.35 ± 0.56*	21.3 ± 2.69^†^	–
**4i**	H	H		19.45 ± 2.39	42.79 ± 1.22^†^	–
**4j**	H	H		16.34 ± 0.63*	38.28 ± 4.36	–
**4k**		OCH_3_	H	41.07 ± 2.33*	80.47 ± 5.08^†^	0.65 ± 0.13
**4l**		OCH_3_	H	33.59 ± 3.29*	76.12 ± 0.88	2.63 ± 0.19
**4m**		OCH_3_	H	12.91 ± 1.27*	69.65 ± 1.52^†^	1.51 ± 0.20

^[a]^Data present here are the mean ± S.E (Table S1) and donepezil as postive control exhibited IC_50_ = 0.079 ± 0.05 µM against AChE and IC_50_ = 10.6 ± 2.1 µM against BChE

*Indicates a significant difference (P < 0.05) in comparison between 4a as unsubstituted derivatives compared with the rest of the compounds in the AChE set

^†^Indicates significant differences (P < 0.05) in comparison between 4a as unsubstituted derivatives compared with the rest of the compounds in the BChE set

To explain the SARs, amino-7,8-dihydro-4H-chromenone derivatives were divided into five categories. First, derivatives **4a–e** were synthesized, where R^2^ and R^3^ was set to H. Among these derivatives, **4a**, the unsubstituted analog, exhibited 21.02% inhibition at 50 µM against AChE. This initial result provided a starting point to investigate the impact of various substitutions at the R^1^ position to enhance potency. Remarkably, improvements in inhibitory potency were observed in all cases containing different groups at R^1^ moiety, indicating that substitutions at the R^1^ position were beneficial for enhancing activity against AChE. The most potent derivatives in this set were **4e** with ethyloxymorpholine substitution, followed by **4b** containing the nitro group. Notably, there was no significant difference in potency between 4-chlorobenzyloxy (**4c**) and 4-bromobenzyloxy (**4d**) derivatives.

The derivatives **4f** and **4g** were developed, with R^1^:H and R^3^:H. Both compounds bearing 4-chlorobenzyloxy (**4f**) and 4-bromobenzyloxy (**4g**) substitutions were nearly inactive, suggesting that substitution at the R^2^ position alone was unfavorable for enhancing inhibitory activity against AChE.

In the subsequent modifications, derivatives **4h–j** (R^1^:H and R^2^:H), bearing 4-chlorobenzyloxy, 3-chlorobenzyloxy, and 4-methoxybenzyloxy substitutions, did not exhibit improved potency compared to compound **4a**. This observation indicated that substitution at the R^3^ position was unfavorable for enhancing inhibitory potency against AChE.

Evaluation of derivatives **4k–m** (R^3^:H and R^2^: OCH_3_) yielded important findings, with compound **4k** (R^1^: 4-fluorobenzyloxy) showing the most potent inhibition at 50 µM with 41.07% inhibition, followed by compound **4l** with 33.59% inhibition. However, it was evident that increasing bulkiness at the R^1^ position (compound **4m**) led to a decrease in potency, as it exhibited only 12.91% inhibition against AChE.

In conclusion, this study revealed valuable insights into the SARs of the 4H- chromenone derivatives against AChE. Substitutions at the R^1^ position alone generally improved potency, with ethyloxymorpholine (**4e**) and the nitro group (**4b**) being particularly effective. On the other hand, substitutions at R^2^ position alone or the R^3^ substitutions with increased bulkiness at the R^1^ position were unfavorable for enhancing inhibitory activity.

### Evaluation of BuChE inhibition

Next, the inhibition of all derivatives against BChE was evaluated, and the results were presented in Table 2.

Among the derivatives, the unsubstituted compound **4a** demonstrated significant inhibition, showing 39.77% activity at 50 µM. Notably, there was a remarkable improvement in inhibitory potency among derivatives **4b-g**, where different moieties were substituted at the R^1^ position. It was observed that both 4-chlorobenzyloxy (**4c**) and 4-bromobenzyloxy (**4d**) substitutions at R^1^ significantly increased the inhibitory activity, with IC_50_ values of 0.89 ± 0.24 µM and 1.19 ± 0.31 µM, respectively. Similarly, derivatives **4e** and **4b** also displayed promising inhibition against BChE. The larger active site of BChE, as compared to AChE, allows for the increased bulkiness of substituents, making these derivatives more favorable for interactions with the BChE active site.

Further examination of derivatives **4f** and **4g**, with substitution at the R^2^ position, revealed improved anti-BChE potency compared to the unsubstituted analog (**4a**). Compound **4f** demonstrated an IC_50_ value of 5.70 ± 0.68 µM, while **4g** exhibited an IC_50_ value of 13.06 ± 2.59 µM. However, **4f** and **4g** recorded lower potency than derivatives **4c** and **4d**, confirming that substitution at the *para* position is favorable for enhancing inhibitory activity against BChE.

Consistent with the observations in AChE, any type of substitution at the R^3^ position (derivatives **4h–j**) was unfavorable for BChE inhibition, resulting in lower potency compared to the substituted analogs. The presence of steric hindrance at the R^3^ position likely played a role in reducing inhibitory activity.

Remarkably, derivatives **4k–m** displayed the best inhibitory potency against BChE, with IC_50_ values ranging from 0.65 ± 0.13 µM to 2.63 ± 0.19 µM. Similar to the findings in AChE evaluations, derivative **4k** was identified as the best analog in this series. It appears that 4-fluorobenzyloxy at the R^1^ position and OCH_3_ at the R^2^ position contribute to improved interactions with the enzyme’s binding site, leading to enhanced inhibitory activity against BChE.

In summary, evaluating the 4H-chromenone derivatives against BChE revealed several potent compounds with encouraging inhibitory activity. Substitutions at the R^1^ and R^2^ positions played pivotal roles in enhancing inhibitory potency, while substitutions at the R^3^ position were generally unfavorable. Derivatives **4k–m** emerged as the most promising analogs regarding BChE inhibition, demonstrating their potential as candidates for further optimization and development as therapeutic agents targeting BChE in conditions such as AD.

### Considering AChE and BChE inhibition

**Fig. 2:**
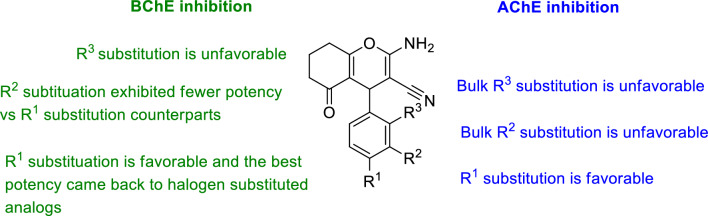
Summary of SARs

Overall, it is evident that the designed backbone of amino chromenone derivatives displayed high potency and selectivity against BChE compared to AChE. A summary of the SARs of AChE and BChE inhibitions is depicted in Fig. 2. For BChE inhibition, the backbone of amino chromenone derivatives showed promising and selective activity in most cases. Notably, substitution at the R^1^ and R^2^ positions favored BChE inhibition. Conversely, substitutions at the R^3^ position resulted in comparatively lower potency, suggesting a less favorable outcome in terms of inhibitory activity. Similarly, the substituents at R^3^ significantly reduce the potency against AChE (Fig. 2).

### Kinetic studies of BChE inhibition

**Fig. 3:**
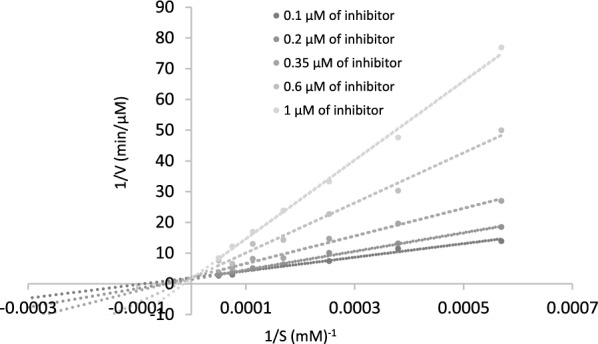
The Lineweaver–Burk plot of the most potent inhibitor **4k** at different concentrations against BChE

The mechanism of inhibition for compound **4k**, identified as the most potent inhibitor of BChE, was investigated through a kinetic study against BChE. The results are illustrated in the reciprocal Lineweaver–Burk plot (Fig. 3). As the concentration of inhibitor **4k** increased, the Michaelis–Menten constant (*K*_*m*_) increased, and the maximum reaction rate (*V*_*max*_) remained unaffected. This behavior indicates that compound **4k** acts as a competitive inhibitor.

**Fig. 4:**
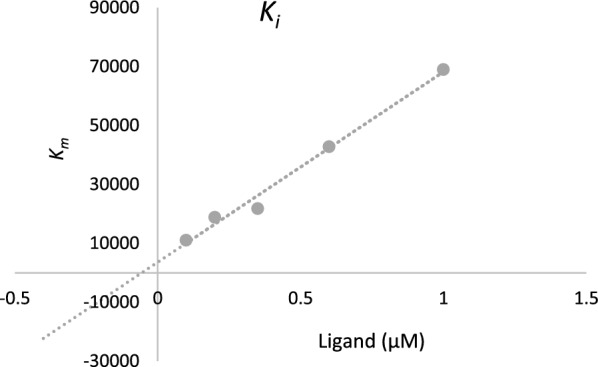
Double reciprocal Lineweaver–Burk plot of **4k** against BChE

Additionally, by plotting the slopes of the lines against various concentrations of the inhibitor, an estimate of the inhibition constant (*K*_*i*_) was obtained, which was determined to be 0.55 µM (R^2^ = 0.9876). The *K*_*i*_ value provides important information about the strength of the interaction between the inhibitor and the enzyme (Fig. 4).

### Docking study

The binding pocket of AChE is approximately 20 Å deep and is composed of the catalytic anionic site (CAS) pocket and includes important residues Glu202, Ser203, and His447 of the catalytic triad. Additionally, the anionic subsite of AChE consists of Trp86. Near the gorge’s entrance is a peripheral anionic subsite (PAS) comprising amino acids Trp86, Tyr337, and Phe338. These residues play crucial roles in substrate binding and catalysis.

**Fig. 5:**
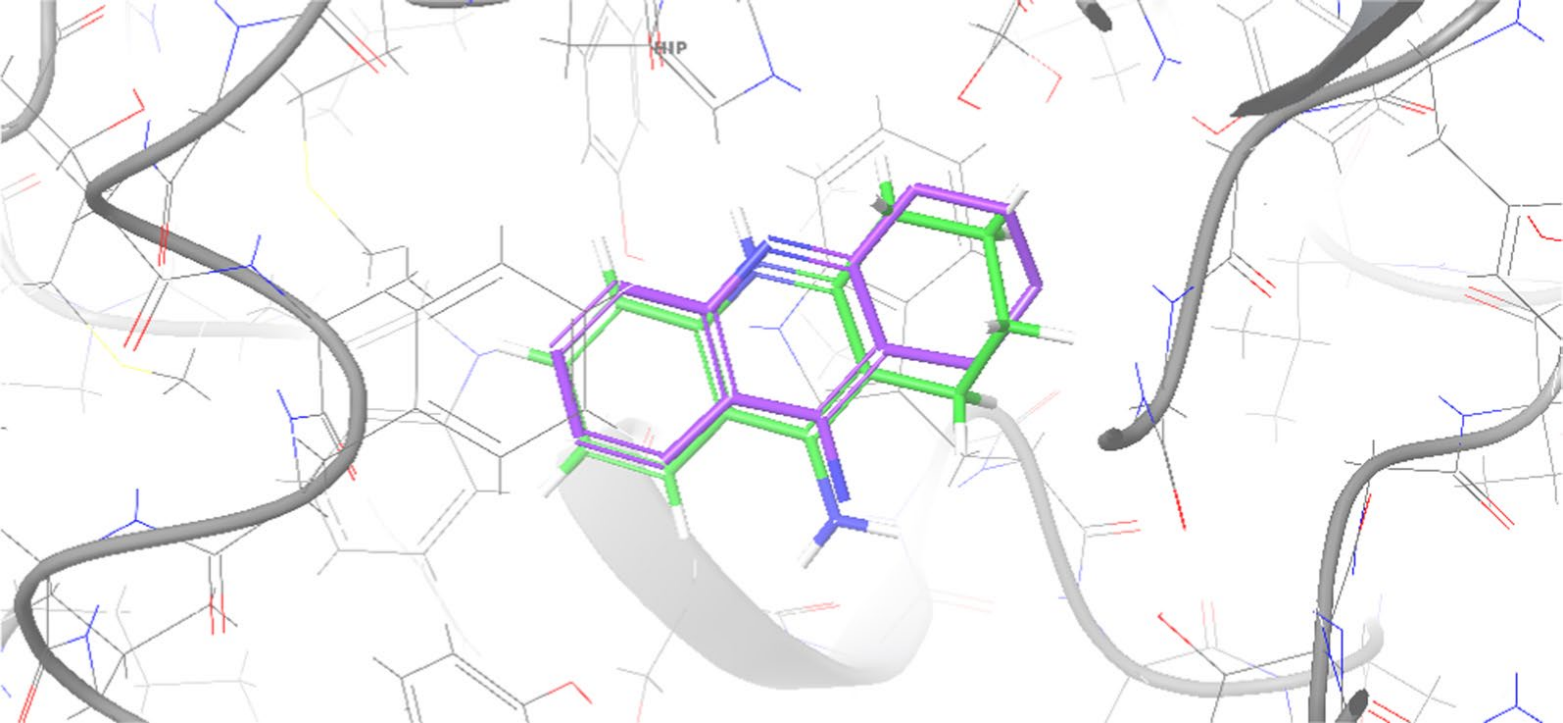
The superimpose structure of crystalographic tacrine (purple) vs docked tacrine (green)

On the other hand, the binding pocket of BChE also contains a catalytic triad, but its composition differs from AChE. In BChE, the catalytic triad of the CAS consists of Ser198, Glu325, and His438. The PAS of BChE includes Asp70 and Tyr332, which are essential for substrate binding and enzymatic activity. Additionally, Trp82 serves as an indicator of the choline-binding site in BChE. The structural differences between the binding pockets of AChE and BChE contribute to their substrate specificity and catalytic activity. Understanding the key residues in these binding pockets is crucial for designing and developing selective inhibitors targeting AChE and BChE for managing AD. As a result, molecular docking was executed to understand the binding mechanism of **4k** as the most potent BChE inhibitor against both the targeted enzymes. Initial validation of molecular docking was performed by the redocking of crystallographic ligands into the active sites of AChE (PDB ID: 4EY7) and BChE (PDB ID: 4BDS) and and the results showed the root-mean-square deviation (RMSD) values less than 2 Å confirming the reliability of the docking procedure (Fig. 5).

**Fig. 6.**
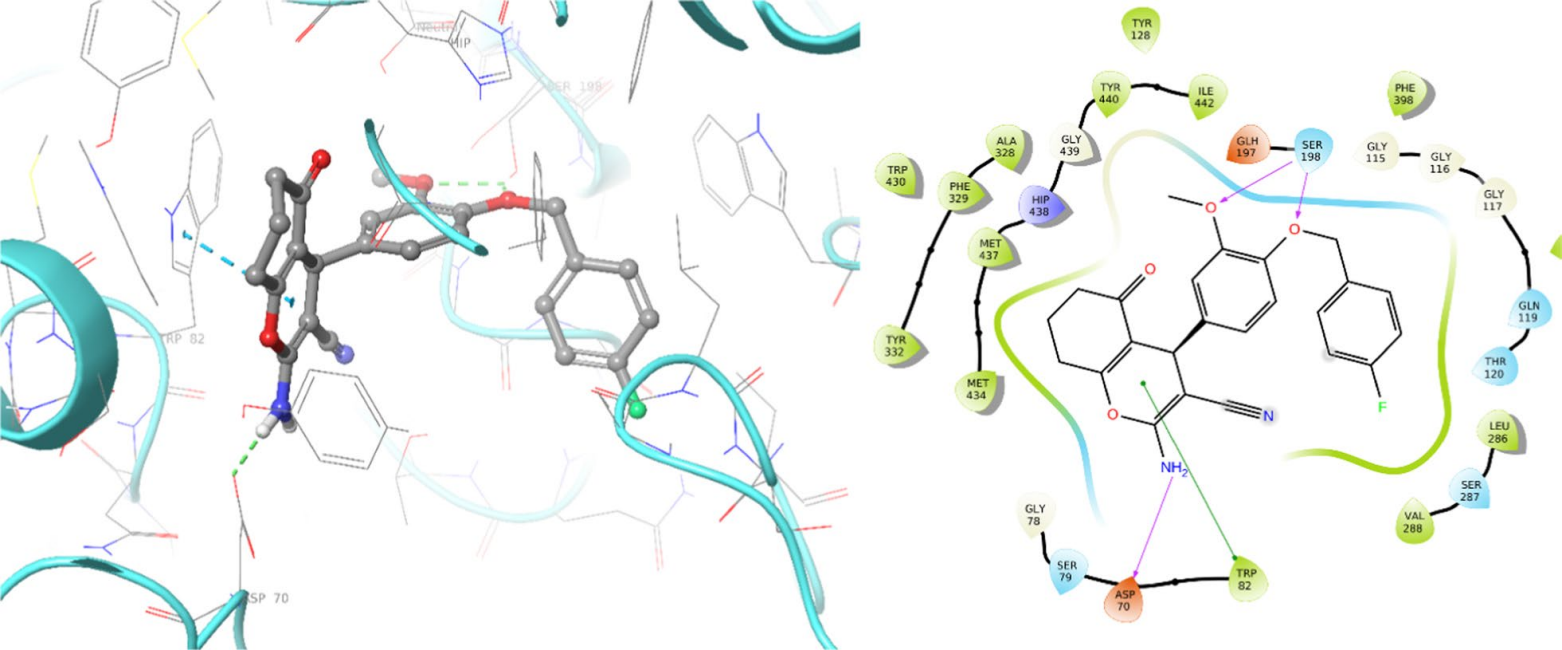
3D and 2D binding model of **4k** within the active site of BChE

The docking results of compound **4k**, identified as the most potent BChE inhibitor, are presented in Fig. 6. The docking analysis revealed important molecular interactions between **4k** and the BChE enzyme. On one side of the molecule, the NH group of chromene-one participated in a hydrogen bond interaction with Asp 70, a crucial residue in the PAS pocket and the chromene-one moiety recorded pi-pi stack interaction with Trp82 of the choline-binding site of BChE. On the other side of the molecule, the OCH_3_ group and the ether linker of **4k** formed two strong hydrogen bond interactions with the critical Ser198 residue of the catalytic triad in the enzyme’s active site (Fig. 6). This interaction is essential for inhibiting the enzymatic activity of BChE.

**Fig. 7:**
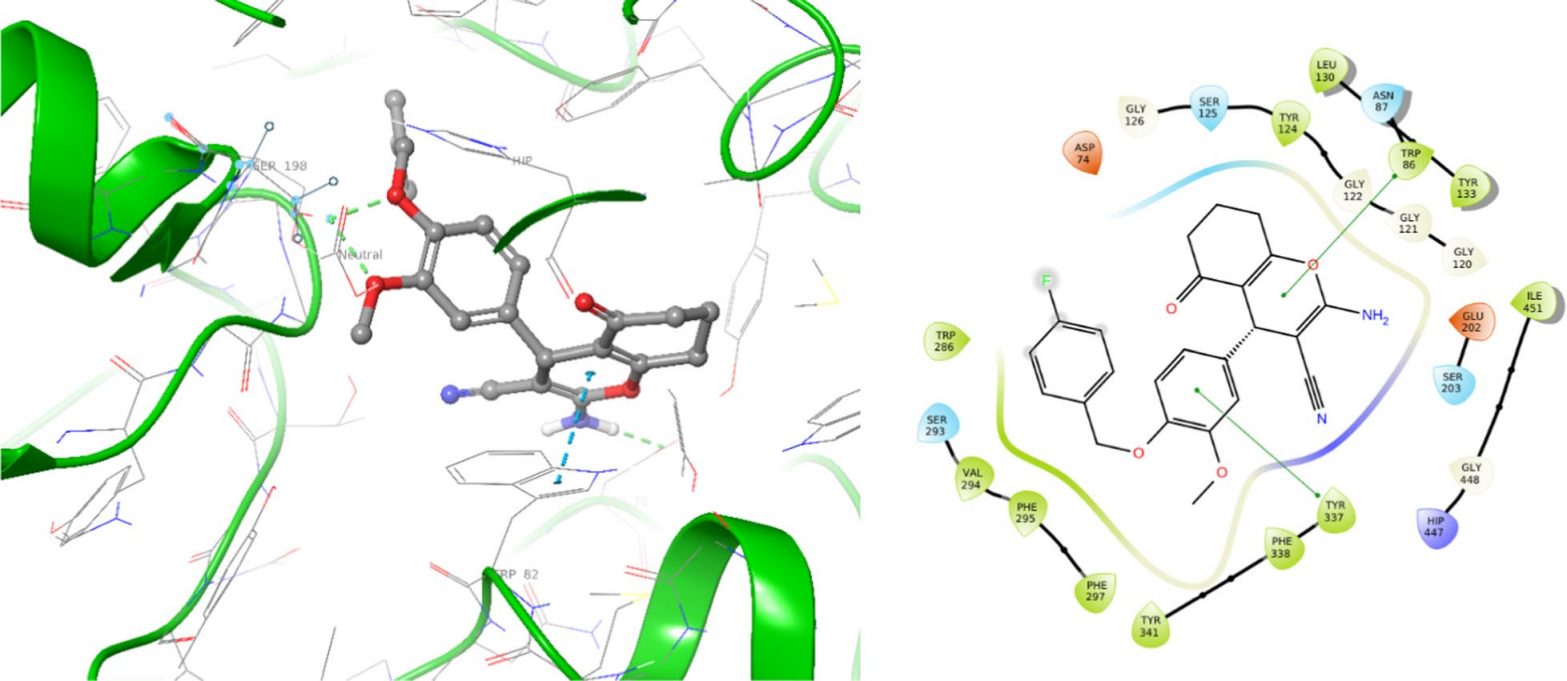
3D and 2D binding model of **4k** within the active site of AChE

Next, the molecular docking study of **4k** as the inactive AChE inhibitor was performed against AChE (Fig. 7). In the AChE binding pocket, compound **4k** exhibited pi-pi stacking interactions with Trp86 and Tyr337 of the PAS. However, it did not show significant interactions with the critical residues of the CAS and the catalytic triad. The absence of strong interactions with the CAS and the catalytic triad explains the low potency of **4k** against AChE. Different studies support that effective interactions with key residues, including those in the PAS and CAS, are crucial for ChE inhibition, and without effective interactions with these key residues, the inhibitor might fail to inhibit the enzymatic activity of AChE effectively [15, 17–20].

### Molecular dynamics simulations

**Fig. 8:**
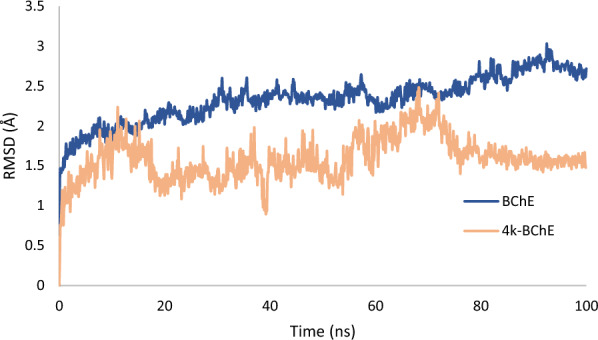
Superimposed RMSD of Cα atoms of BChE in complex with **4k** (red) and BChE (blue)

Molecular Dynamics (MD) simulations were performed to comprehend the intricate dynamic behaviors and structural transitions exhibited by the **4k**-BChE complex compared to its unbound apoenzyme state. The simulation procedures were done by employing Schrödinger’s Desmond software, adhering to an established procedural documented in the previous scientific literature [21]. Throughout the MD simulation, the RMSD trajectory of the **4k**-BChE complex exhibited fluctuations up to 20 nanoseconds. Then recorded, stability up to 55 ns at the value of 1.5 Å followed by fluctuations. Gradually, these oscillations converged into a stable equilibrium state from 76 ns that persisted throughout the simulation, characterized by an RMSD value of 1.52 Å. In contrast, the RMSD values for the apoenzyme displayed a gradual increase up and then reached a steady-state equilibrium with an RMSD value of 2.5 Å. The observations strongly suggest that the **4k**-BChE complex boasts stability compared to its unbound apoenzyme counterpart (Fig. 8).

**Fig. 9:**
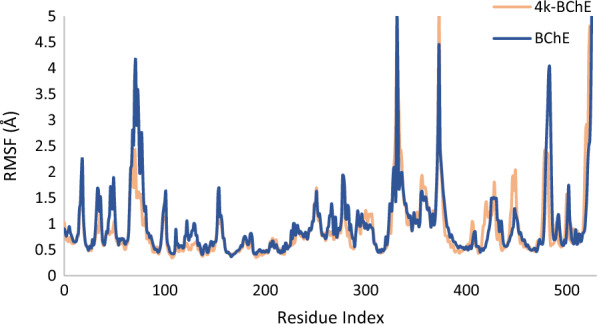
RMSF graph of the BchE (blue) and BChE in complex with **4k** (orange)

Root mean square fluctuation (RMSF) plots of the protein were subjected to a comprehensive analysis of the robustness of the protein’s structure and degree of mobility. A comparative examination of protein fluctuations was conducted on unbound apo form and **4k**-BChE. This analysis identified residues that are crucial in interacting with the enzyme. As depicted in Fig. 9, the RMSF values were reduced within the complex, particularly in regions such as the PAS domain (Asp70, Tyr 332), and choline binding site (Trp82). These areas were carefully determined as the key regions undergoing structural modifications upon complex formation (Fig. 9).

This evidence strongly implies that the binding of compound **4k** with the protein triggers noteworthy alterations in the flexibility of these specific regions, indicative of their active involvement in the interactions with the enzyme. These findings shed light on the pivotal role played by these regions in stabilizing the complex and guiding its functional dynamics.

In Fig. 9, residues exhibiting fluctuations exceeding 2 angstroms are also observed, which is a common characteristic in enzymes. This phenomenon is particularly notable for Gln67-Gly75 located at the entrance of the enzyme binding site, Val331-Tyr332, as well as Tyr373- Trp376 situated in unbounded regions, thus their higher fluctuation is acceptable. Additionally, Pro480-Asn485, positioned in the flap region of the enzyme, typically experiences increased fluctuations. Furthermore, it is well-established that the N and C terminals of enzymes often display higher fluctuations compared to other regions. Therefore, the elevated fluctuation observed in residues Arg520 to Val529 can be attributed to their location within these terminal regions, thus justifying their higher fluctuation levels.

**Fig. 10:**
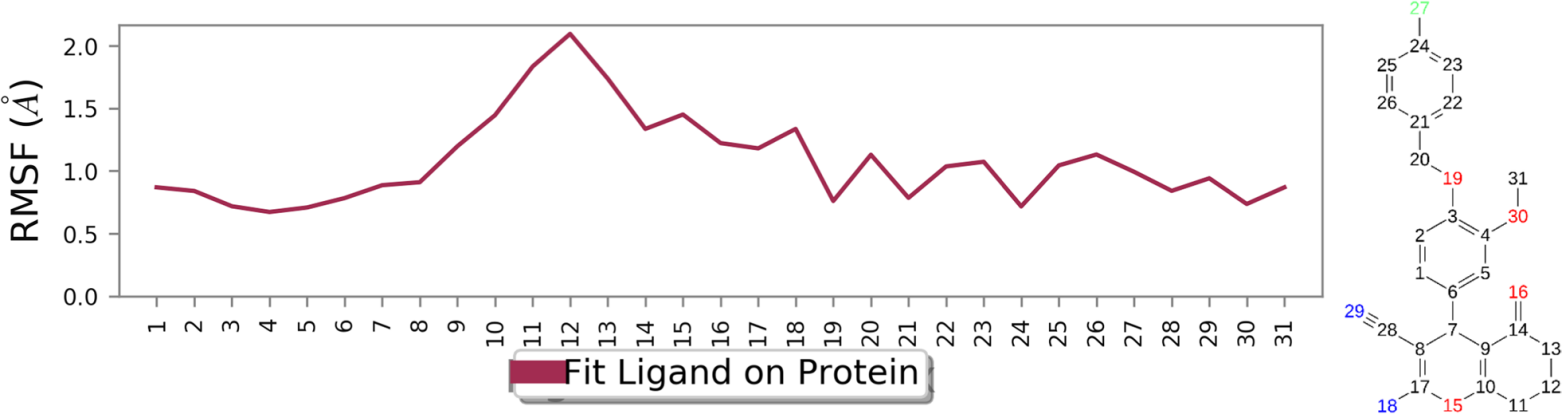
RMSF graph of **4k** in the active site

RMSF of the ligand is depicted in Fig. 10, and this data provides valuable insights into the nature of interactions between each ligand atom and the protein. The graphical representation elucidates that all atoms of the ligand positioned within the active site of BChE exhibit RMSD values below 2 Å. The fewer value affirming the formation of consistent and favorable binding interactions. This unequivocally confirms the presence of interactions between the ligand and the enzyme. These observations strongly suggest that the ligand establishes and maintains stable interactions with the active site of the BChE enzyme.

**Fig. 11:**
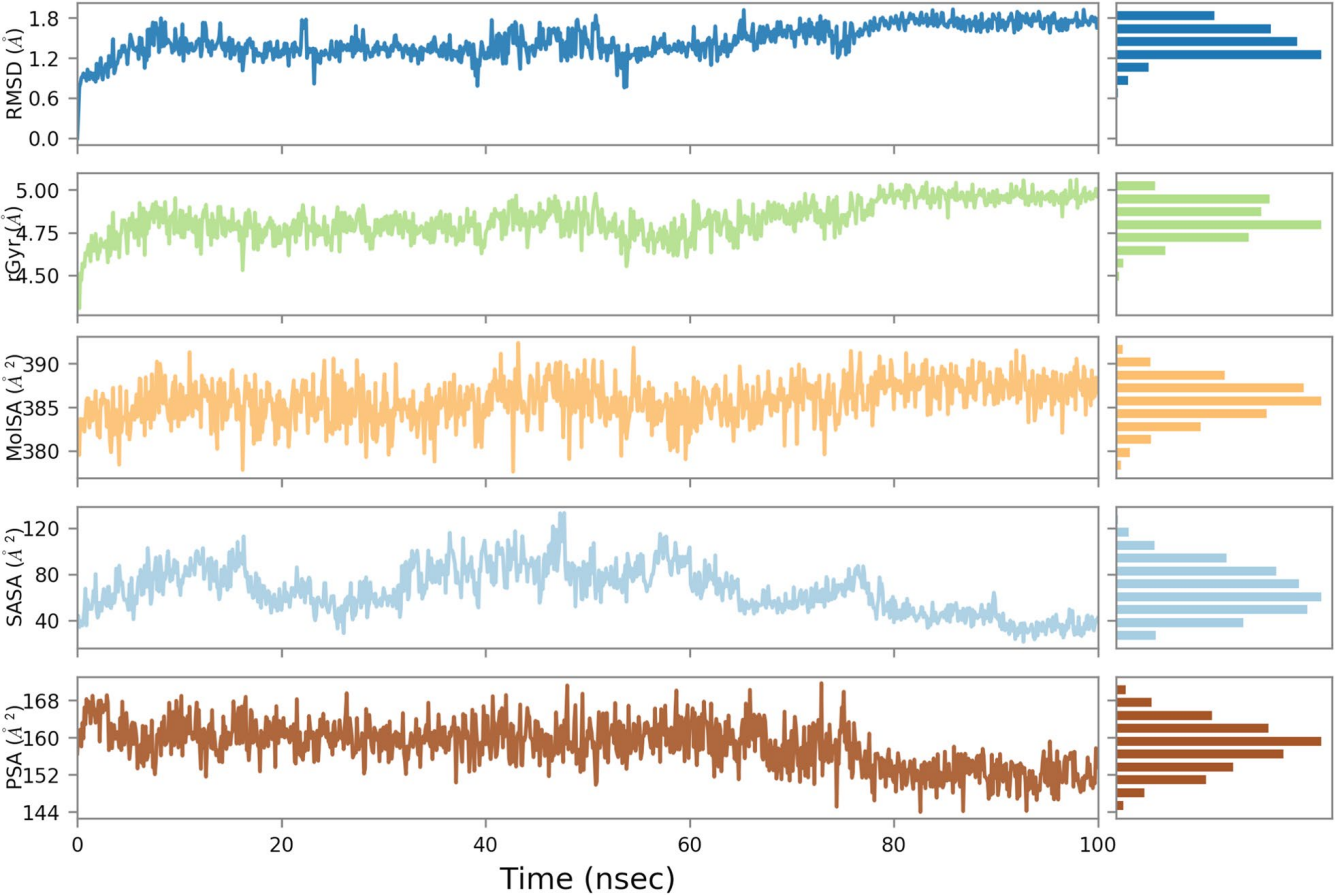
Ligand property trajectory for the **4k**-BChE complex

Continuing our investigation, we delved into the properties of the ligand, encompassing crucial parameters such as Molecular Surface Area (MolSA), Radius of Gyration (rGyr), Polar Surface Area (PSA), and Solvent Accessible Surface Area (SASA). These properties provided a deeper understanding of the ligand’s structural characteristics and its interactions within the complex. Results are exhibited in Fig. 11.

The rGyr served as an indicator of the ligand’s overall conformational spread. The rGyr values ranged from approximately 4.0 to 5.0 Å, with an equilibrium value of around 4.40 Å up to 80 ns followed by an increase to 5 and stable till the end of the study. These values indicated the extent of ligand extension, with higher rGyr values suggesting a more extended conformation. Moving on to MolSA, which corresponds to the van der Waals surface area, we observed values ranging from 376 to 388 Å^2^, converging at an equilibrium value of 385 Å^2^. This parameter sheds light on the ligand’s spatial occupation and its interactions within the complex. The SASA parameter, representing the surface area accessible to a water molecule, exhibited a range of 35 to 130 Å^2^, with an average value of 80 Å^2^. This metric quantified the extent to which the ligand was exposed to the surrounding solvent environment. Additionally, the PSA, which quantifies polar groups on the ligand’s surface, yielded an average value of 160 Å^2^. This parameter offered insights into the ligand’s potential for forming hydrogen bonds and other polar interactions.

**Fig. 12:**
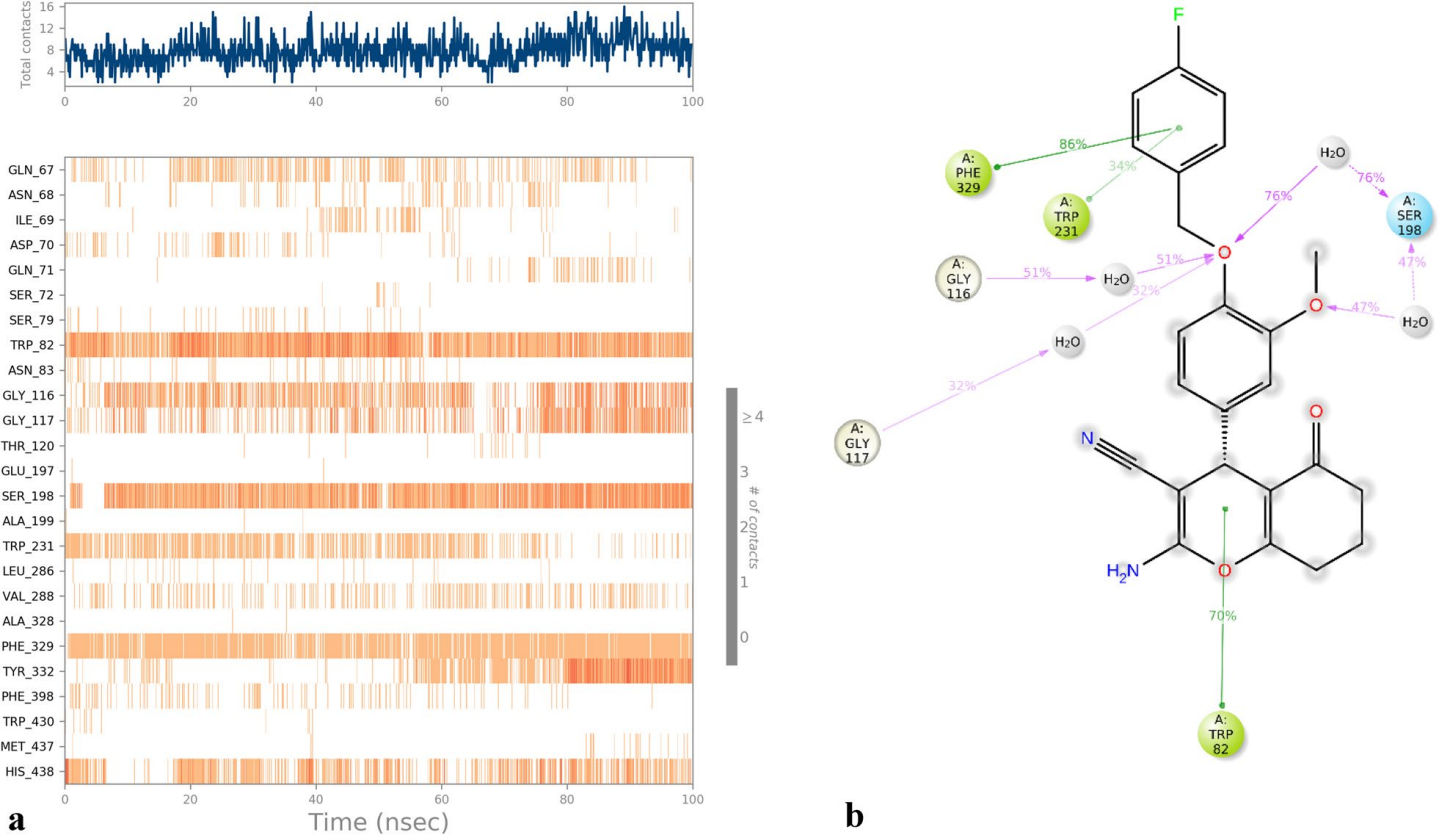
**a** Timeline representation of the interactions and contacts. **b** A schematic of detailed ligand interactions with the protein residues that occur more than 30.0% of the simulation time

The interaction between the protein and the **4k** is visually represented in Fig. 12a, where the evidence of these interactions was observed during MD simulation. In most cases, three interactions emerge in each nanosecond. Throughout the majority of the MD run, these interactions demonstrated their persistence. Specifically, Trp82, Ser198 Phe329, and His438 were identified as key participants engaging with the **4k** in these interactions. Figure 12b exhibited a schematic of detailed ligand interactions with the protein residues. The chromenone ring showcased an intriguing pi-pi stacking interaction with Trp82 (70%). Furthermore, the benzyloxy ring of the **4k** prominently participated in three hydrogen bonding interactions with Ser198 (76%), Gly117 (32%), Gly116 (51%) mediated with water. The 2-methoxy substituted on the benzyloxy ring also participates in hydrogen bonding interaction with Ser198 using water as the intermediary. The 4-fluro benzyl illustrated two significant pi-pi stacking interactions with Trp231 (34%) and Phe329, accounting for 34% and 86% of the simulation time, respectively.

A comparison of the results of binding interactions from molecular docking (Fig. 6) versus those observed in MD (Fig. 12a) highlighted that the chromenone ring participated in a pi-pi stacking interaction with Trp82. Additionally, interactions were seen between Ser198 and the OCH_3_ group, as well as the ether linker of **4k.**

Our study showed that substitutions at the R^1^ and R^2^ positions were crucial for enhancing inhibitory potency, while substitutions at the R^3^ position were generally unfavorable. Specifically, derivatives **4k** emerged as the most promising analogs for BChE inhibition, with persistent interactions involving residues Trp82, Ser198, Phe329, and His438 throughout most of the MD simulations.

#### ADMET properties and in silico toxicity

Table 3 presents the drug-likeness properties according to Lipinski’s rule of five, which specifies that a promising drug candidate should not exceed more than five hydrogen bond donors or ten hydrogen bond acceptors, its molecular weight should be below 500 Dalton, and its logP should not exceed 5. Pan-assay interference compounds (PAINS) are chemical compounds that often give false-positive results in high-throughput screens. As outlined, all the studied compounds followed these favorable drug-likeness standards [22].

**Table 3:** Drug-likeness prediction for **4a-m**

Compound	Molecular Weight (Dalton)	LogP	Rotatable Bonds	Acceptors	Donors	Surface Area (Angstrom)	PAINS alert
**4a**	266.3	2.50	1	4	1	173.54	0
**4b**	311.29	2.41	2	6	1	131.35	0
**4c**	406.87	4.73	4	5	1	173.54	0
**4d**	451.32	4.84	4	5	1	177.10	0
**4e**	395.46	2.21	5	7	1	169.87	0
**4f**	406.87	4.73	4	5	1	169.87	0
**4g**	451.32	4.84	4	5	1	177.10	0
**4h**	406.87	4.73	4	5	1	173.54	0
**4i**	406.87	4.73	4	5	1	173.54	0
**4j**	402.45	4.09	5	6	1	174.71	0
**4k**	420.44	4.22	5	6	1	178.88	0
**4l**	420.44	4.22	5	6	1	178.88	0
**4m**	436.90	4.74	5	6	1	185.01	0

Moreover, Table 4 demonstrates the ADMET profile—an abbreviation for Absorption, Distribution, Metabolism, Excretion, and Toxicity—correlating with pharmaceutical substances’ behaviors in biological systems calculated using the pkCSM and SwissADME online [23]. For the compounds under consideration, the predicted Human Intestinal Absorption (HIA) values suggest a high probability of effective absorption through the gastrointestinal lining. It is also projected that the compounds exhibit a level of Caco-2 permeability that is conducive to oral ingestion. Metabolically, it is significant to highlight that the compounds are unlikely to inhibit CYP2D6, an enzyme a trait that is deemed beneficial. Furthermore, the substances display toxicity levels within acceptable ranges, qualifying them as viable for subsequent phases of drug development and optimization.

**Table 4:** ADMET prediction of the synthesized **4a-m**

Compd	Absorption		Distribution ^b^		Metabolism ^b^				Excretion	Toxicity		
	HIA%^a^	Caco2 permeability	VDss (log L/Kg)^b^	BBB permeability	CYP3A4 inhibition	CYP2D6 inhibition	CYP2C9 inhibition	CYP2C19 inhibition	Total Clearance	Oral Rat Acute Toxicity (LD_50_)	Oral Rat Chronic Toxicity (LOAEL)	Hepatotoxicity
**4a**	94.967	0.485	0.279	− 0.009	No	No	No	No	0.358	2.52	1.416	No
**4b**	90.284	1.017	0.177	− 0.553	No	No	No	No	0.373	2.593	1.873	No
**4c**	93.421	0.485	0.344	− 0.474	Yes	No	Yes	Yes	− 0.297	2.655	1.7	No
**4d**	93.353	0.857	0.358	− 0.483	Yes	No	Yes	Yes	− 0.318	2.666	1.689	No
**4e**	86.268	1.115	0.554	− 0.614	No	No	No	No	0.832	2.866	0.392	No
**4f**	93.633	0.479	0.383	− 0.475	Yes	No	Yes	Yes	− 0.286	2.637	1.669	No
**4g**	93.566	0.471	0.397	− 0.484	Yes	No	Yes	Yes	− 0.307	2.647	1.659	No
**4h**	93.719	0.48	0.343	− 0.479	Yes	No	Yes	Yes	− 0.164	2.595	1.674	No
**4i**	93.719	0.48	0.343	− 0.479	Yes	No	Yes	Yes	− 0.164	2.595	1.674	No
**4j**	100	0.709	0.215	− 0.539	Yes	No	Yes	Yes	0.408	2.535	1.768	No
**4k**	99.164	0.719	0.056	− 0.736	Yes	No	Yes	Yes	0.189	2.678	1.616	No
**4l**	99.164	0.733	0.056	− 0.736	Yes	No	Yes	Yes	− 0.736	2.697	1.616	No
**4m**	99.257	0.713	0.207	− 0.703	Yes	No	Yes	Yes	− 0.155	2.752	1.578	No

^a^HIA (Human Intestinal Absorption): > 80% is high and < 30% is poor; ^b^VDss (steady-state volume of distribution): log L/Kg: > 0.45 is high and < − 0.15 is low

## Conclusion

In summary, we successfully designed and synthesized a series of amino-7,8-dihydro-4H-chromenone derivatives (**4a–m**), which were subsequently evaluated for their inhibitory activity against AChE and BChE. Most compounds exhibited noteworthy BChE inhibitory activity and compounds **4k** and **4c** emerged as particularly potent BChE inhibitors, with IC_50_ values of 0.65 ± 0.13 µM and 0.89 ± 0.24 µM, respectively, compared with the positive control donepezil (IC_50_ = 10.6 ± 2.1 µM). Furthermore, a thorough kinetic analysis unveiled that compound **4k** acts as a competitive inhibitor, with a *K*_*i*_ value of 0.55 µM. The interaction profiles of the most potent compound within the active sites of AChE and BChE were explored through molecular docking. These investigations illuminated that the **4k** established substantial interactions within the BChE active site, distinguishing their behavior from that within the AChE active site. This unity between computational predictions and experimental results lends credibility to our findings. Furthermore, molecular dynamics study was performed on the **4k**-BChE and the apoenzyme. This investigation showcased that our compound **4k** attains a favorable conformation within the BChE active site, effectively occupying crucial enzyme pockets such as the PAS and CAS. This comprehensive exploration enhances our understanding of how our compound interacts with the enzyme and provides insights into its mode of action. Overall, our findings underscore the potential of these designed compounds as promising candidates for therapeutic interventions targeting AD.

## Methods and materials

### Chemistry

All chemicals were purchased from Sigma-Aldrich (USA) and used without further purification. The infrared spectra of the products were recorded on a Bruker FT-IR Spectrometer using KBr as a matrix. Proton and carbon NMR spectra of the novel compounds were recorded on a NMR FT- 300 and 75 MHz spectrometer (Bruker) in DMSO-d_6_ solution. Melting points were taken on a Keison Electrothermal IA9100 Melting Point apparatus fixed at 1 °C / min. Elemental analyses of compounds for C, H and N were performed using a Heraeus CHN-O-S Rapid analyzer.

#### General procedure for the synthesis of benzaldehyde derivatives 2c–m

Compounds **2c–m** were prepared by minor modifications in previous our work. In a 50 mL round bottom flask equipped with magnet and 10 mL CH_3_CN, 4-hydroxy-3-methoxybenzaldehyde, 2-hydroxybenzaldehyde, *meta* or *para*-hydroxybenzaldehyde (1 mmol) along with K_2_CO_3_ (2 mmol) was added and stirred at room temperature for 5 min. After that, appropriated benzyl halides or 4-(2-chloroethyl)morpholin-4-ium chloride (1.2 mmol) in CH_3_CN (5 mL) in a dropwise manner was added to the latter suspension and the final reaction mixture was stirred under reflux condition to complete reaction. The progress of the reaction was monitored with TLC (silica gel plat, GF_25_, Merck). Then, this reaction mixture was poured into crushed ice and filtered off. The crud products were recrystallized in ethanol to give corresponding benzaldehydes. Ethyl acetate extraction was also used for liquid aryl aldehydes [15].

#### General procedure for the synthesis of 4H-chromene derivatives 4a-m

For synthesis of 4H-chromene derivatives **4**, at first, 1.5 mmol of malononitrile **1**, 1 mmol of substituted benzaldehyde **2** and 0.03 g of sodium dihydrogen phosphate as a catalyst was poured in the ethanol (10 mL) solvent in a 50 cc flask equipped with a magnet. The mixture was stirred for 2 to 4 h. Then, 1,3-cyclohexanedione **3** (1 mmol) and additional sodium dihydrogen phosphate (0.03 g) were added to the system, and the mixture was kept under reflux conditions for 4 to 6 h. The completion of the reaction was checked by thin layer chromatography. After that, water was added to the latter mixture and products were filtered off and recrystallized in ethanol to give pure products **4a–m** (Scheme 1) and Table 1, Additional file 1.

*2-Amino-5-oxo-4-phenyl-5,6,7,8-tetrahydro-4H-chromene-3-carbonitrile (4a)* Yield 88%; mp 214–215 °C; IR (KBr, cm^−1^): ν_max_ 3322 (N–H), 3168, 2920, 2191(CN), 1683 (C=C), 1651, 1453,

1368, 1261, 1209, 1065, 1000, and 699 [16].

*2-Amino-4-(4-nitrophenyl)-5-oxo-5,6,7,8-tetrahydro-4H-chromene-3-carbonitrile (4b)* Yield 90%; mp 235–236 °C; IR (KBr, cm^−1^): ν_max_ 3415 (N–H), 3335, 3216, 2914, 2195 (CN), 1681 (C=C), 1650, 1518, 1345, 1261, 1209, 1068, 1005, 821, and 695 [16].

*2-Amino-4-{4-[(4-chlorobenzyl)oxy]phenyl}-5-oxo-5,6,7,8-tetrahydro-4Hchromene-3-carbonitrile (4c)* Yield 90%; mp 183–185 °C; IR (KBr, cm^−1^): ν_max_ 3437 (N–H), 3326, 3046, 2954, 2874, 2206, 1685 (C=C), 1601, 1508, 1406, 1361, 1197, 996, and 822; ^1^H NMR (DMSO-*d*_*6*_, 300 MHz) δ (ppm): 7.51–7.44 (4H,m,CHAr), 7.10 (2H, *d, J* = 9 Hz, CHAr), 7.00 (2H, s, NH2), 6, 93(2H, *d, J* = 9 Hz, CHAr), 5.08 (2H, s, OCH2), 4.17 (1H, s, CH), 2.63–2.60 (2H, m, CH2), 2.32–2.22 (2H, m, CH2), and 1.99–1.84 (2H, m, CH2); ^13^C NMR (DMSO-*d*_*6*_, 75 MHz) δ (ppm): 196.3, 164.6, 158.8, 157.3, 137.7, 136.7, 132.8, 129.9, 128.9, 128.7, 120.3, 115.0, 114.5, 68.8, 58.8, 36.8, 35.1, 26.9, and 20.2; Anal. calcd for C_23_H_19_ClN_2_O_3_: C, 67.90; H, 4.71; N, 6.89; Found: C,67.81; H, 4.80; N, 6.98%.

*2-Amino-4-{4-[(4-bromobenzyl)oxy]phenyl}-5-oxo-5,6,7,8-tetrahydro-4Hchromene-3-carbonitrile (4d)* Yield 90%; mp 188–190 °C; IR (KBr, cm^−1^): ν_max_ 3440 (N–H), 3328, 3044, 2954, 2885, 2204, 1685 (C=C), 1665, 16.00, 1508, 1362, 1223, 1197, 1066, 996, and 799; ^1^H NMR (DMSO-*d6*, 300M*Hz*) δ (ppm): 7.62 (2H, d, *J* = 9Hz CHAr), 7.43 (2H, d, *J* = 9Hz CHAr), 7.11 (2H, d, *J* = 9Hz CHAr), 7.00 (2H, s, NH_2_), 6.94 (2H, d, *J* = 9Hz CHAr), 5.06 (2H, s, OCH_2_), 4.16 (1H, s, CH), 2.67–2.62 (2H, m, CH_2_), 2.38–2.20 (2H, m, CH_2_), and 2.01–1.84 (2H, m, CH_2_); ^13^C NMR(DMSO-*d6*, 75 MHz) δ (ppm): 196.4, 164.7, 158.9, 157.4,, 137.8, 137.2, 131.8, 130.2, 128.7, 121.4, 120.4, 115.0, 114.5, 68.9, 58.8, 26.9, and 20.3; Anal. calcd for C_23_H_19_BrN_2_O_3_: C, 61.21; H, 4.24; N, 6.21; Found: C, 61.01; H, 4.12; N, 6.39%.

*2-Amino-4-[4-(2-morpholinoethoxy)phenyl]-5-oxo-5,6,7,8-tetrahydro-4Hchromene-3-carbonitrile(4e)* Yield 70%; mp 188–190 °C; IR (KBr, cm^−1^): ν_max_ 3419 (N–H), 3337, 3011, 2951, 2193, 1678 (C=C), 1652, 1609, 1510, 1458, 1372, 1239, 1123, 1039, 915, and 860; ^1^H NMR (DMSO-*d*_*6*_, 300M*Hz*) δ (ppm): 7.09 (2H, d, *J* = 9Hz CHAr), 6.99 (2H, s, NH_2_), 6.88 (2H, d, *J* = 9Hz CHAr), 4.15 (1H, s, CH), 4.06 (2H, t, *J* = 6Hz, OCH_2_), 3.59 (4H, t, *J* = 6Hz, 2 OCH_2_), 2.69 (2H, t, *J* = 6Hz, NCH_2_),

2.62 (2H, brs, CH_2_), 2.48(4H, t, *J* = 6Hz, 2 NCH_2_), 2.38–2.01 (2H, m, CH_2_), and 2.00–1.84 (2H, m, CH_2_); ^13^C NMR(DMSO-*d*_*6*_, 75 MHz) δ (ppm): 196.3, 164.6, 158.9, 157.6, 137.4, 128.7, 120.3, 114.7, 114.6, 66.6, 65.7, 58.9, 57.5, 54.1, 36.8, 35.1, 26.9, and 20.3; Anal. calcd for C_22_H_25_N_3_O_4_: C, 66.82; H, 6.37; N, 10.63; C, 66.71; H, 6.18; N, 10.89%.

*2-Amino-4-{3-[(4-chlorobenzyl)oxy]phenyl}-5-oxo-5,6,7,8-tetrahydro-4H-chromene-3-carbonitrile (4f)* Yield 87%; mp 214–216 °C; IR (KBr, cm^−1^): ν_max_ 3319 (N–H), 3168, 3045, 2929, 2877, 2195, 1681 (C=C), 1645, 1606, 1484, 1450, 1366, 1273, 1213, 1040, 1001, 851, and 810; ^1^H NMR (DMSO-*d*_*6*_, 300 M*Hz*) δ (ppm): 7.52–7.45 (4H, m, CHAr), 7.23 (1H, t, *J* = 9Hz, CHAr), 7.05 (2H, s, NH_2_), 6.86 (1H, dd, 1*J* = 9Hz, 2*J* = 3Hz, CHAr), 6.80–6.75 (2H, m, CHAr), 5.07 (2H, s, OCH_2_), 4.19 (1H, s, CH), 2.68–2.59 (2H, m, CH_2_), 2.38–2.20 (2H, m, CH_2_), and 2.02–1.82 (2H, m, CH_2_); ^13^C NMR(DMSO-*d*_*6*_, 75 MHz) δ (ppm): 196.3, 165.0, 158.9, 158.7, 146.9, 136.5, 132.8, 130.1, 129.9, 128.9, 120.3, 120.2, 114.4, 114.1, 112.8, 68.8, 58.4, 36.7, 35.7, 26.9, and 20.2; Anal. calcd for C_23_H_19_ClN_2_O_3_: C, 67.90; H, 4.71; N, 6.89; Found C, 67.74; H, 4.60; N, 6.97%.

*2-Amino-4-{3-[(4-bromobenzyl)oxy]phenyl}-5-oxo-5,6,7,8-tetrahydro-4Hchromene-3-carbonitrile (4 g)* Yield 86%; mp 224–226 °C; IR (KBr, cm^−1^): ν_max_ 3317 (N–H), 3164, 3046, 2930, 2877, 2194, 2930, 2877, 2194, 1681 (C=C), 1645, 1607, 1487, 1450, 1367, 1271, 1214, 1068, 1039, 1001, 851, and 806; ^1^H NMR (DMSO-*d*_*6*_, 300 MHz) δ (ppm): 7.62 (2H, d, *J* = 9 Hz CHAr), 7.45 (2H, d, *J* = 9 Hz CHAr), 7.23 (1H, t, *J* = 9 Hz CHAr), 7.04 (2H, s, NH_2_), 6.86 (1H, dd,* J* = 3 Hz and *J* = 9 Hz CHAr), 6.77 (2H, m, CHAr), 5.06 (2H, s, OCH_2_), 4.19 (1H, s, CH), 2.68–2.60 (2H, m, CH_2_), 2.38–2.20 (2H, m, CH_2_), and 2.02–1.84 (2H, m, CH_2_); ^13^C NMR(DMSO-*d*_*6*_, 75 MHz) δ (ppm): 196.3, 165.0, 158.9, 158.7, 146.9, 136.5, 132.8, 130.1, 129.9, 128.9, 120.3, 120.2, 114.4, 114.1, 112.8, 68.8, 58.4, 36.7, 35.7, 26.9, and 20.2;Anal. calcd for C_23_H_19_BrN_2_O_3_: C, 61.21; H, 4.24; N, 6.21; Found: C, 61.29; H, 4.04; N, 6.43%.

*2-Amino-4-{2-[(4-chlorobenzyl)oxy]phenyl}-5-oxo-5,6,7,8-tetrahydro-4H-chromene-3-carbonitrile (4h)* Yield 90%; mp 224–226 °C; IR (KBr, cm^−1^): ν_max_ 3332, 3190, 2920, 2192, 1603 (C=C), 1648, 1451, 1364 (C–N), 1251, 1211, 1074, 534; ^1^H NMR (DMSO-*d*_*6*_, 300 M*Hz*) δ (ppm): 7.58 (2H, d, *J* = 9 Hz CHAr), 7.50 (2H, d, *J* = 9 Hz CHAr), 7.19–7.14 (1H, m, CHAr), 7.08–6.99(2H, m, CHAr), 6.92 (1H, d, *J* = 9 Hz CHAr), 6.86 (2H, s, NH_2_), 5.17 (1H, d, *J* = 12 Hz, OCH), 5.06 (1H, d, *J* = 12 Hz, OCH), 4.58 (1H, s, CH), 2.48–2.30 (2H, m, CH_2_), 2.28–2.16 (2H, m, CH_2_), and 1.98–1.73 (2H, m, CH_2_); ^13^C NMR (DMSO-*d*_*6*_, 75 MHz) δ (ppm): 196.2, 165.1, 159.1, 156.1, 136.7, 133.0, 132.7, 130.1, 129.4, 128.8, 121.2, 120.4, 113.5, 112.9, 69.2, 57.9, 40.8, 36.8, 30.8, 26.8 and 20.3; Anal. calcd for C_23_H_19_ClN_2_O_3_: C, 67.90; H, 4.71; N, 6.89; Found: C,67.71; H, 4.60; N, 6.98%.

*2-Amino-4-{2-[(3-chlorobenzyl)oxy]phenyl}-5-oxo-5,6,7,8-tetrahydro-4H-chromene-3-carbonitrile(4i)* Yield 85%; mp 204–207 °C; IR (KBr, cm^−1^): ν_max_ 3323 (N–H), 3216, 2948, 2188, 1603 (C=C), 1653, 1453, 1371, 1255, 1211, 1069, 701; ^1^H NMR (DMSO-*d*_*6*_, 300 MHz) δ(ppm): 7.63 (1H, s, CHAr) 7.53–7.40 (3H, m, CHAr), 7.19–6.88 (4H, m, CHAr), 6.85 (2H, s, NH_2_), 5.20 (1H, d, *J* = 12Hz, OCH), 5.12 (1H, d, *J* = 12Hz, OCH), 4.64 (1H, s, CH), 2.66–2.47 (2H, m, CH_2_), 2.36–2.20 (2H, m, CH_2_), and 2.03–187 (2H, m, CH_2_); ^13^C NMR (DMSO-*d*_*6*_, 75 MHz) δ (ppm): 196.2, 165.2, 159.1, 155.9, 140.3, 133.5, 133.7, 130.7, 128.2, 128.1, 127.8, 126.7, 121.3, 120.4, 113.6, 112.8, 69.2, 58.0, 36.8, 30.4, 26.8 and 20.3; Anal. calcd for C_23_H_19_ClN_2_O_3_: C, 67.90; H, 4.71; N, 6.89; Found: C, 67.68; H, 4.60; N, 6.93%.

*2-Amino-4-{2-[(4-methoxybenzyl)oxy]phenyl}-5-oxo-5,6,7,8-tetrahydro-4H-chromene-3-carbonitrile(4j)* Yield 75%; mp 215–218 °C; IR (KBr, cm^−1^): ν_max_ 3332 (N–H), 3220, 2943, 2201, 1608 (C=C), 1652, 1449, 1370, 1241, 1215, 827, 700; ^1^H NMR (DMSO-*d*_*6*_, 300 MHz) δ (ppm): 7.49 (2H, d, *J* = 9Hz CHAr), 7.19–7.01 (3H, m, CHAr), 7.00 (2H, d, *J* = 9Hz CHAr), 6.90 (1H, t, *J* = 9Hz CHAr), 6.84 (2H, s, NH_2_), 5.07 (1H, d, *J* = 12Hz, OCH), 4.98 (1H, d, *J* = 12Hz, OCH), 4.55 (1H, s, CH), 3.79 (3H, s, OCH_3_), 2.49–2.44 (2H, m, CH_2_), 2.27–2.17 (2H, m, CH_2_), and 1.95–1.83 (2H, m, CH_2_); ^13^C NMR (DMSO-*d*_*6*_, 75 MHz) δ (ppm): 196.2, 165.1, 159.4, 159.1, 156.4, 132.9, 130.0, 129.6, 129.3, 128.1, 120.9, 120.4, 114.2, 113.5, 112.9, 69.8, 57.9, 55.5, 36.8, 30.9, 26.8 and 20.3; Anal. calcd for C_24_H_22_N_2_O_4_: C, 71.63; H, 5.51; N, 6.96; Found: C,71.89; H, 5.45; N, 7,09%.

*2-Amino-4-{4-[(4-fluorobenzyl)oxy]-3-methoxyphenyl}-5-oxo-5,6,7,8-tetrahydro-4H-chromene-3-carbonitrile (4k)* Yield 90%; mp 227–230 °C; IR (KBr, cm^−1^): ν_max_ 3322 (N–H), 3207, 2935, 2188, 1650 (C=C), 1674, 1459, 1363, 1271, 828, 536; ^1^H NMR (DMSO-*d*_*6*_, 300 MHz) δ (ppm): 7.50 (2H, dd, *J* = 9Hz, *J* = 6Hz, CHAr), 7.28–7.20 (2H, m, CHAr), 7.00 (2H, s, NH_2_), 6.97 (1H, t, *J* = 9Hz CHAr), 6.78 (1H, d, *J* = 3Hz), 6.67 (1H, dd,* J* = 6Hz, *J* = 3Hz, CHAr), 5.03 (2H, s, OCH_2_), 4.18 (1H, s, CH), 3.76 (3H, s, OCH_3_), 2.69–2.60 (2H, m, CH_2_), 2.39–2.29 (2H, m, CH_2_), and 2.25–1.88 (2H, m, CH_2_); ^13^C NMR (DMSO-*d*_*6*_, 75 MHz) δ (ppm): 196.4, 164.7, 163.8, 160.6, 158.9, 149.2, 146.9, 138.3, 134.0, 133.9, 130.5, 130.4, 120.3, 119.4, 115.8, 115.5, 114.3, 114.1, 111.9, 69.7, 58.7, 56.0, 35.3, 26.9 and 20.3; Anal. calcd for C_24_H_21_FN_2_O_4_: C, 68.56; H, 5.03; N, 6.66; Found: C, 68.74; H, 4.91; N, 6.81%.

*2-Amino-4-{4-[(3-fluorobenzyl)oxy]-3-methoxyphenyl}-5-oxo-5,6,7,8-tetrahydro-4H-chromene-3-carbonitrile (4l)* Yield 85%; mp 191–194 °C; IR (KBr, cm^−1^): ν_max_ 3300 (N–H), 3183, 2924, 2186, 1599 (C=C), 1656, 1464, 1364, 1254, 1035, 940, 683; ^1^H NMR (DMSO-*d*_*6*_, 300 MHz) δ (ppm): 7.46 (1H, m, CHAr), 7.30–7.14 (3H, m, CHAr), 6.99 (2H, s, NH_2_), 6.96 (1H, t, *J* = 9Hz CHAr), 6.79 (1H, d, *J* = 3Hz), 6.65 (1H, dd,* J* = 6Hz, *J* = 3Hz, CHAr), 5.08 (2H, s, OCH_2_), 4.18 (1H, s, CH), 3.78 (3H, s, OCH_3_), 2.70–2.60 (2H, m, CH_2_), 2.34–2.25 (2H, m, CH_2_), and 2.04–1.90 (2H, m, CH_2_); ^13^C NMR (DMSO-*d*_*6*_, 75 MHz) δ (ppm): 196.4, 164.7, 164.2, 161.0, 158.9, 149.2, 146.8, 140.8, 138.5, 130.9, 123.9, 120.3, 119.4, 115.1, 114.8, 111.9, 69.6, 58.7, 56.0, 39.1, 35.3, 26.9 and 20.3; Anal. calcd for C_24_H_21_FN_2_O_4_; C, 68.56; H, 5.03; N, 6.66; Found: C, 68.40; H, 4.99; N, 687%.

*A-amino-4-{4-[(3-chlorobenzyl)oxy]-3-methoxyphenyl}-5-oxo-5,6,7,8-tetrahydro-4H-chromene-3-carbonitrile (4m)* Yield 90%; mp 201–204 °C; IR (KBr, cm^−1^): ν_max_ 3316 (N–H), 3207, 2936, 2187, 1599 (C=C), 1656, 1461, 1363, 1253, 1133, 772; ^1^H NMR (DMSO-*d6*, 300 MHz) δ (ppm): 7.52 (1H, s, CHAr), 7.47–7.38 (3H, m, CHAr), 7.00 (2H, s, NH_2_), 6.96 (1H, d, *J* = 9Hz CHAr), 6.80 (1H, d, *J* = 3Hz, CHAr), 6.66 (1H, dd,* J* = 6Hz, *J* = 3Hz, CHAr), 5.07 (2H, s, OCH_2_), 4.18 (1H, s, CH), 3.77 (3H, s, OCH_3_), 2.74–2.55 (2H, m, CH_2_), 2.39–2.25 (2H, m, CH_2_), and 2.02–1.85 (2H, m, CH_2_); ^13^C NMR (DMSO-*d*_*6*_, 75 MHz) δ (ppm): 196.4, 164.8, 158.9, 149.3, 146.8, 140.4, 138.5, 133.5, 130.8, 128.2, 127.8, 126.6, 120.3, 119.4, 114.3, 111.9, 69.6, 58.7, 56.0, 35.3, 26.9 and 20.3; Anal. calcd for C_24_H_21_ClN_2_O_4_; C, 65.98; H, 4.85; N, 6.41; Found: C, 65.93; H, 4.88; N, 6.44%.

### Screening of AChE and BChE inhibitory activity

Cholinesterase inhibitory activities of all derivatives were assessed using the modified Ellman’s method [24]. Briefly, 20 µL AChE 0.18 units/mL, or 20 µL BChE 0.162 units/mL, and 20 µL DTNB (301 μM) were added to 200 μL sodium phosphate buffer (0.1 mol/L, pH = 7.4) in separate wells of a 96-well microplate and gently mixed. Then, 10 μL of different concentrations of test compounds were added to each well and incubated for 15 min at 37 °C followed by the addition of acetylthiocholine (ATCh) or butyrylthiocholine (BTCh) (20 μL, final concentration of 452 μM). The absorbance of each well was measured at 415 nm using a microplate reader. IC_50_ and inhibition values were calculated with the software curve expert as the mean of three independent experiments and expressed as mean ± SEM [25, 26].

### Enzyme kinetic studies

The inhibitory mode of the most potent compound, **4k**, was investigated against BChE. The study involved enzymatic assays using different substrate concentrations butyrylthiocholine (0.1–1 mM) and varying concentrations of the inhibitors. A Lineweaver–Burk plot was generated to determine the inhibition type and calculate the Michaelis–Menten constant (*K*_*m*_). Secondary plots were constructed to determine the experimental inhibitor constant (*K*_*i*_). These analyses aimed to understand the inhibitory mechanisms of **4k**, their affinity for the enzymes, and their potential as therapeutic agents for conditions related to cholinesterase activity.

### Molecular docking

The induced fit docking (IFD) evaluations were performed according to previously reported procedures [25, 27].

### MD simulation

The MD simulation in this study was performed using the Schrodinger 2018‐4 suite [28]. The initial pose for the MD simulation was obtained through the IFD method and the pose is uploaded in a public repository (https://zenodo.org/records/10600816). The protein–ligand complex was solvated with explicit water molecules (SPC model) and placed in an orthorhombic box with appropriate dimensions under Periodic Boundary Conditions to set up the MD system. Counterions and a 0.15 M NaCl solution were added to neutralize the system and mimic physiological ionic concentrations. The MD protocol consisted of minimization, pre-production, and production MD steps. In the minimization step, the system was allowed to relax for 2500 steps using the steepest descent algorithm. The temperature was gradually increased from 0 to 300 K with a small force constant applied to the enzyme to prevent abrupt changes. MD simulation was carried out in the NPT ensemble (constant number of atoms, constant pressure of 1.01325 bar, and constant temperature of 300 K). The Nose–Hoover chain thermostat with a 1.0 ps interval and the Martyna-Tobias-Klein barostat with a 2.0 ps interval were used for temperature and pressure control using an isotropic coupling style. Long-range electrostatic forces were calculated using the Particle-mesh-based Ewald method with a cutoff radius of 9.0 Å for Columbic forces.

The MD simulations of the protein–ligand complexes were conducted for 100 ns. The systems’ structural changes and dynamic behavior were analyzed by calculating RMSD, RMSF and examining the interaction diagrams [29].

### Statistical analysis

Statistical analyses and graphical presentations were conducted using GraphPad Prism version 9 software (GraphPad Software, Inc.). The difference among the groups was analyzed using a one-way analysis of variance (ANOVA) test followed by Tukey post hoc tests. Statistical significance differences were P < 0.05.

### Supplementary Information

**Additional file 1.** **Fig. S1.** ^1^H NMR spectrum of product **4c**. **Fig. S2.** ^13^CNMR spectrum of product **4c**. **Fig. S3.** ^1^H NMR spectrum of product **4d**. **Fig. S4.** ^13^CNMR spectrum of product **4d**. **Fig. S5.** ^1^H NMR spectrum of product **4e**. **Fig. S6.** ^13^CNMR spectrum of product **4e**. **Fig. S7.** ^1^H NMR spectrum of product **4f**. **Fig. S8.** ^13^CNMR spectrum of product **4f**. **Fig. S9**. ^1^H NMR spectrum of product **4g**. **Fig. S10.** ^13^CNMR spectrum of product **4g**. **Fig. S11.** ^1^H NMR spectrum of product **4h**. **Fig. S12.** ^13^CNMR spectrum of product **4h**. **Fig. S13.** ^1^H NMR spectrum of product **4i**. **Fig. S14.** ^13^CNMR spectrum of product **4i**. **Fig. S15.** ^1^H NMR spectrum of product **4j**. **Fig. S16.** ^13^CNMR spectrum of product **4j**. **Fig. S17.** ^1^H NMR spectrum of product **4k**. **Fig. S18.** ^13^CNMR spectrum of product **4k**. **Fig. S19.** ^1^H NMR spectrum of product **4l**. **Fig. S20.** ^13^CNMR spectrum of product **4l**. **Fig. S21.** ^1^H NMR spectrum of product **4m**. **Fig. S22.** ^13^CNMR spectrum of product** 4m**

## Data Availability

The datasets generated and/or analyzed during the current study are available in the Worldwide ProteinData Bank with PDB ID of 4EY7 and 4BDS repository.
