# Supplementary material for: Amino-7,8-dihydro-4H-chromenone derivatives as potential inhibitors of acetylcholinesterase and butyrylcholinesterase for Alzheimer’s disease management; in vitro and in silico study
Source: BMC Chem. 2024 Apr 10;18(1):70. doi: 10.1186/s13065-024-01170-x (PMC11007943; doi:10.1186/s13065-024-01170-x)
Supplement: Supplementary file 1 — Additional file 1. Fig. S1. 1H NMR spectrum of product 4c. Fig. S2. 13CNMR spectrum of product 4c. Fig. S3. 1H NMR spectrum of product 4d. Fig. S4. 13CNMR spectrum of product 4d. Fig. S5. 1H NMR spectrum of product 4e. Fig. S6. 13CNMR spectrum of product 4e. Fig. S7. 1H NMR spectrum of product 4f. Fig. S8. 13CNMR spectrum of product 4f. Fig. S9. 1H NMR spectrum of product 4g. Fig. S10. 13CNMR spectrum of product 4g. Fig. S11. 1H NMR spectrum of product 4h. Fig. S12. 13CNMR spectrum of product 4h. Fig. S13. 1H NMR spectrum of product 4i. Fig. S14. 13CNMR spectrum of product 4i. Fig. S15. 1H NMR spectrum of product 4j. Fig. S16. 13CNMR spectrum of product 4j. Fig. S17. 1H NMR spectrum of product 4k. Fig. S18. 13CNMR spectrum of product 4k. Fig. S19. 1H NMR spectrum of product 4l. Fig. S20. 13CNMR spectrum of product 4l. Fig. S21. 1H NMR spectrum of product 4m. Fig. S22. 13CNMR spectrum of product 4m [file 13065_2024_1170_MOESM1_ESM.pdf]

Supporting information

2-Amino-4-{4-[(4-chlorobenzyl)oxy]phenyl}-5-oxo-5,6,7,8-tetrahydro-4Hchromene-3-carbonitrile (4c)

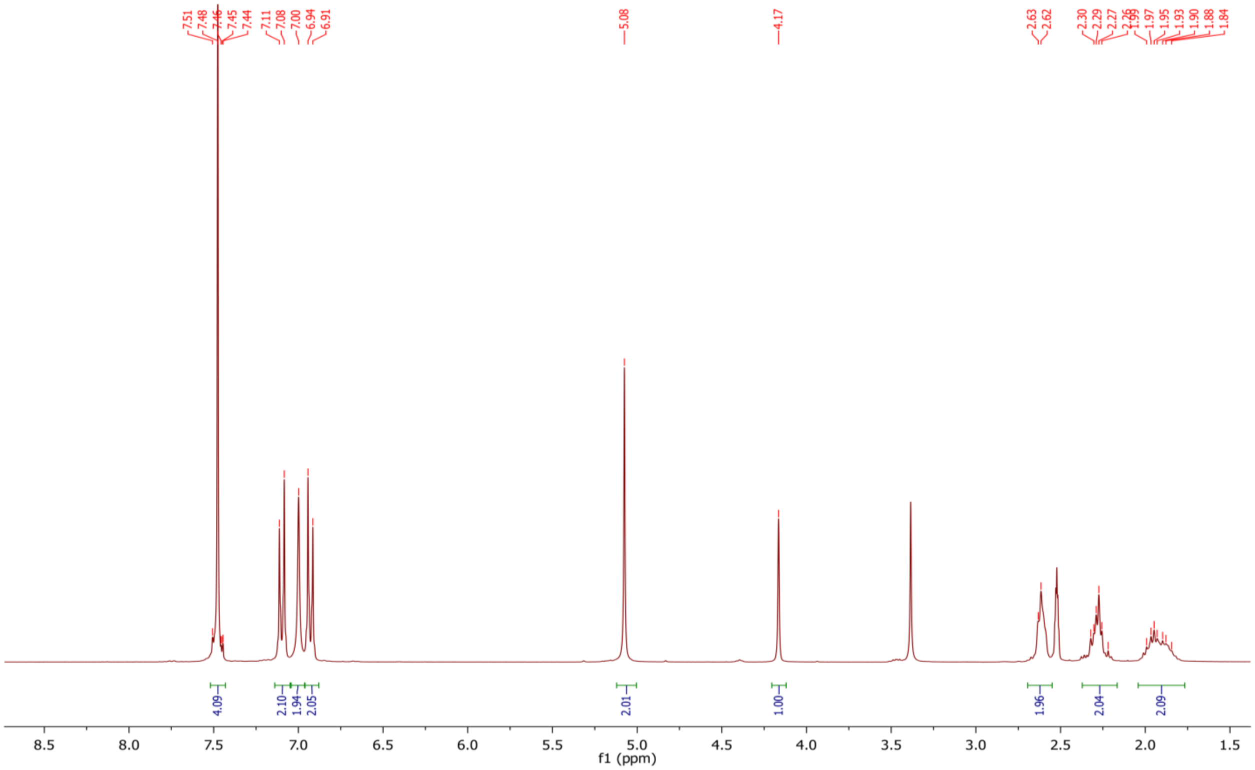

Fig. S1. ^{1}H NMR spectrum of product **4c**

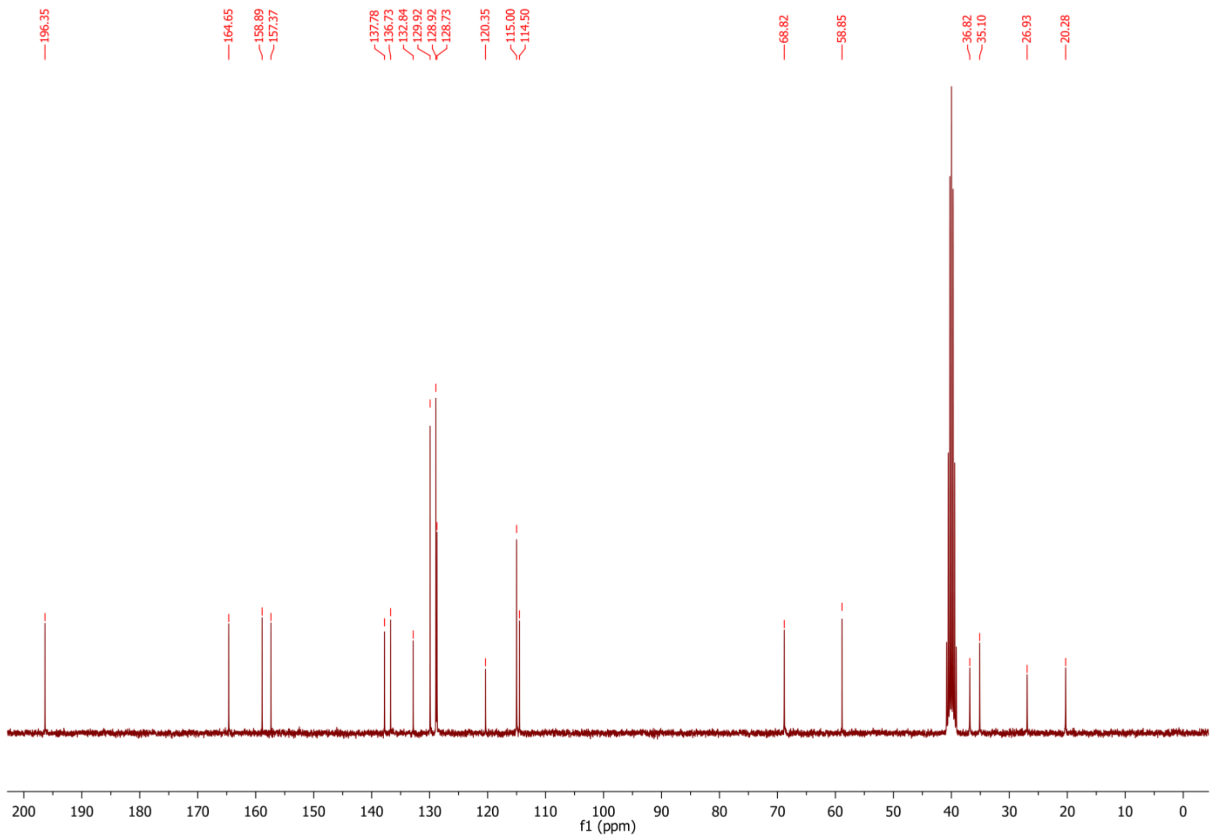

Fig. S2. ^{13}CNMR spectrum of product **4c**

Supporting information

2-Amino-4-{4-[(4-bromobenzyl)oxy]phenyl}-5-oxo-5,6,7,8-tetrahydro-4Hchromene-3-carbonitrile (4d)

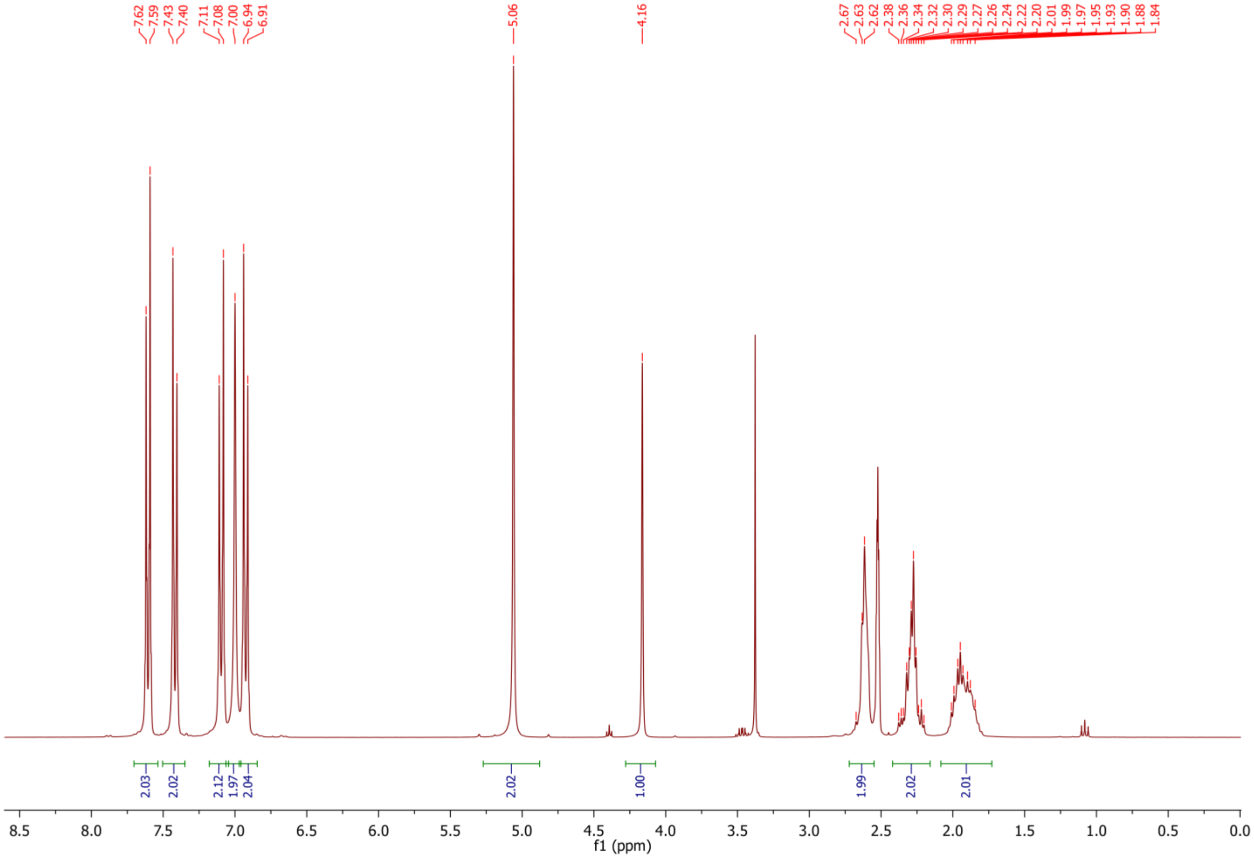

Fig. S3. ^{1}H NMR spectrum of product **4d**

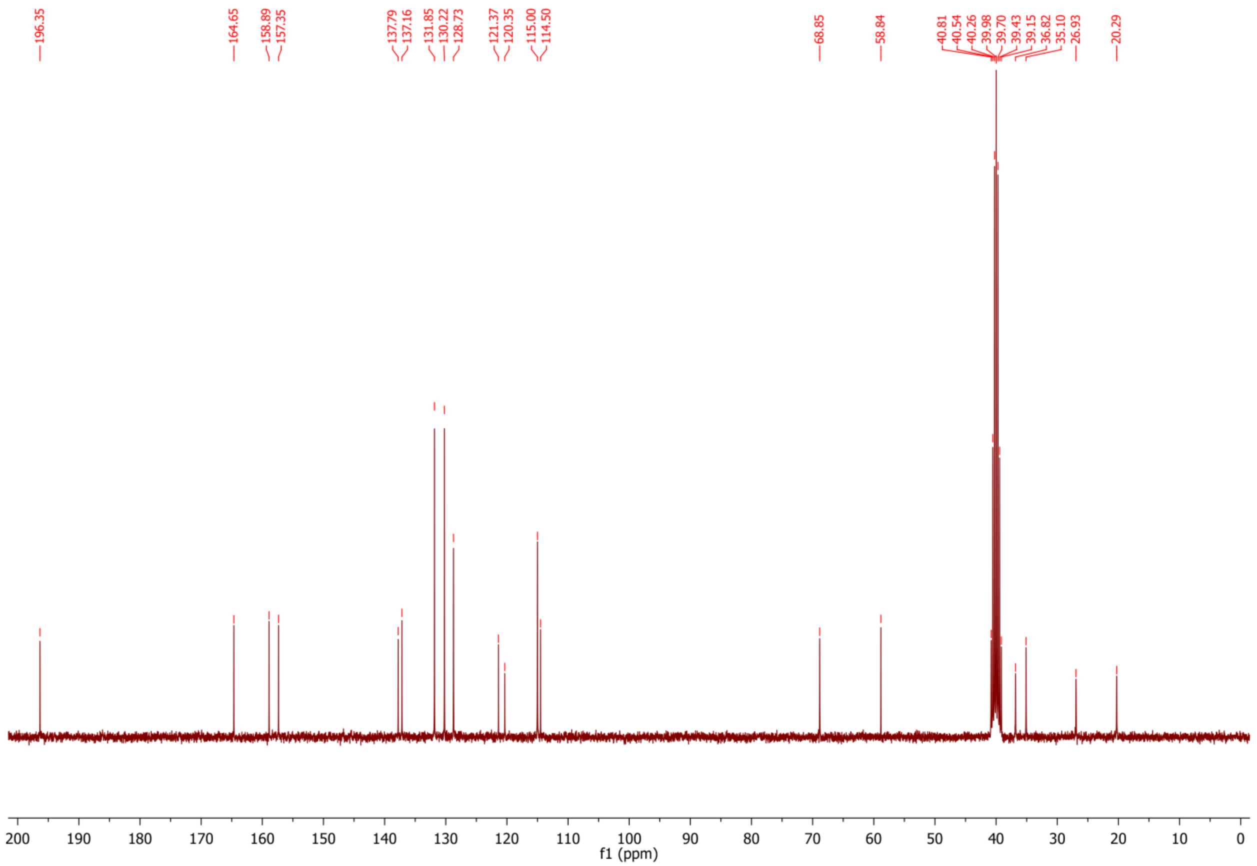

Fig. S4. ^{13}CNMR spectrum of product **4d**

2-Amino-4-[4-(2-morpholinoethoxy)phenyl]-5-oxo-5,6,7,8-tetrahydro-4Hchromene-3-carbonitrile(4e)

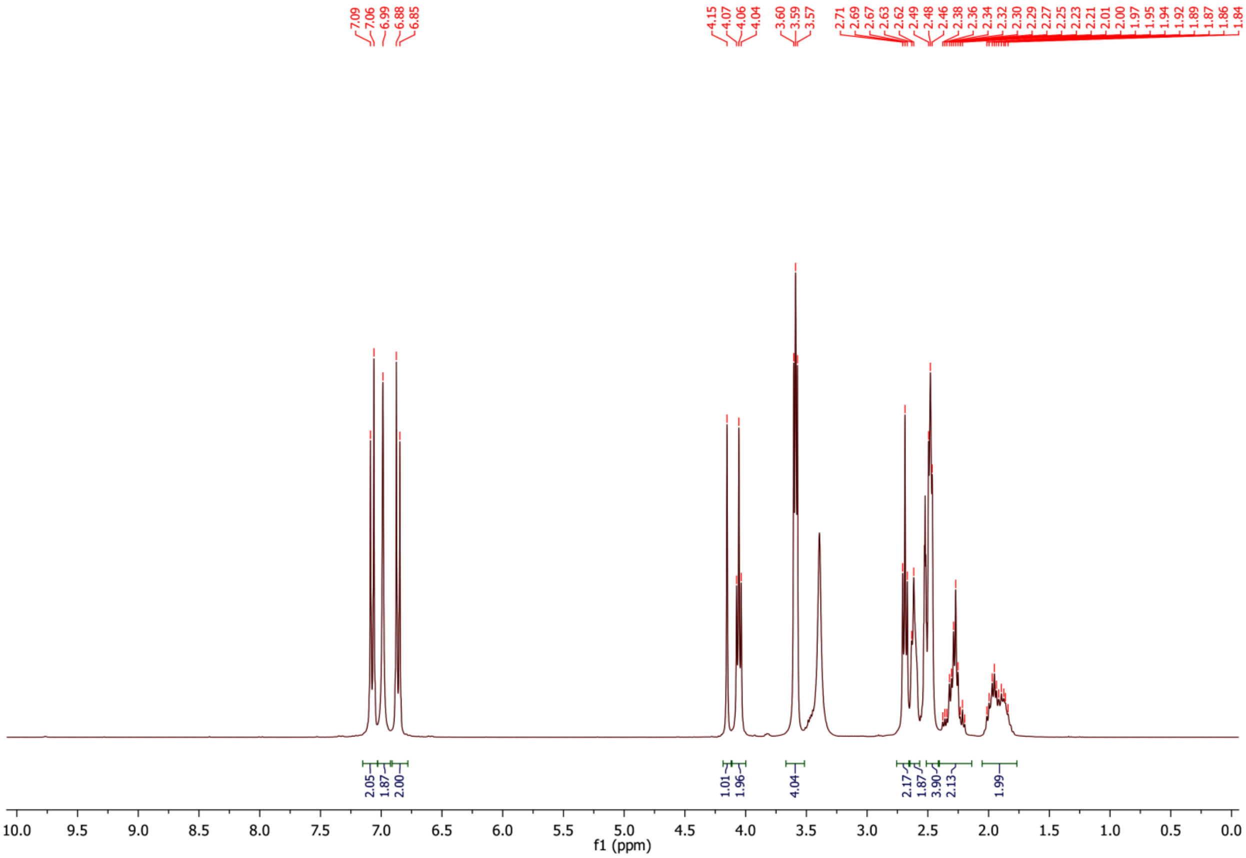

Fig. S5. ^{1}H NMR spectrum of product **4e**

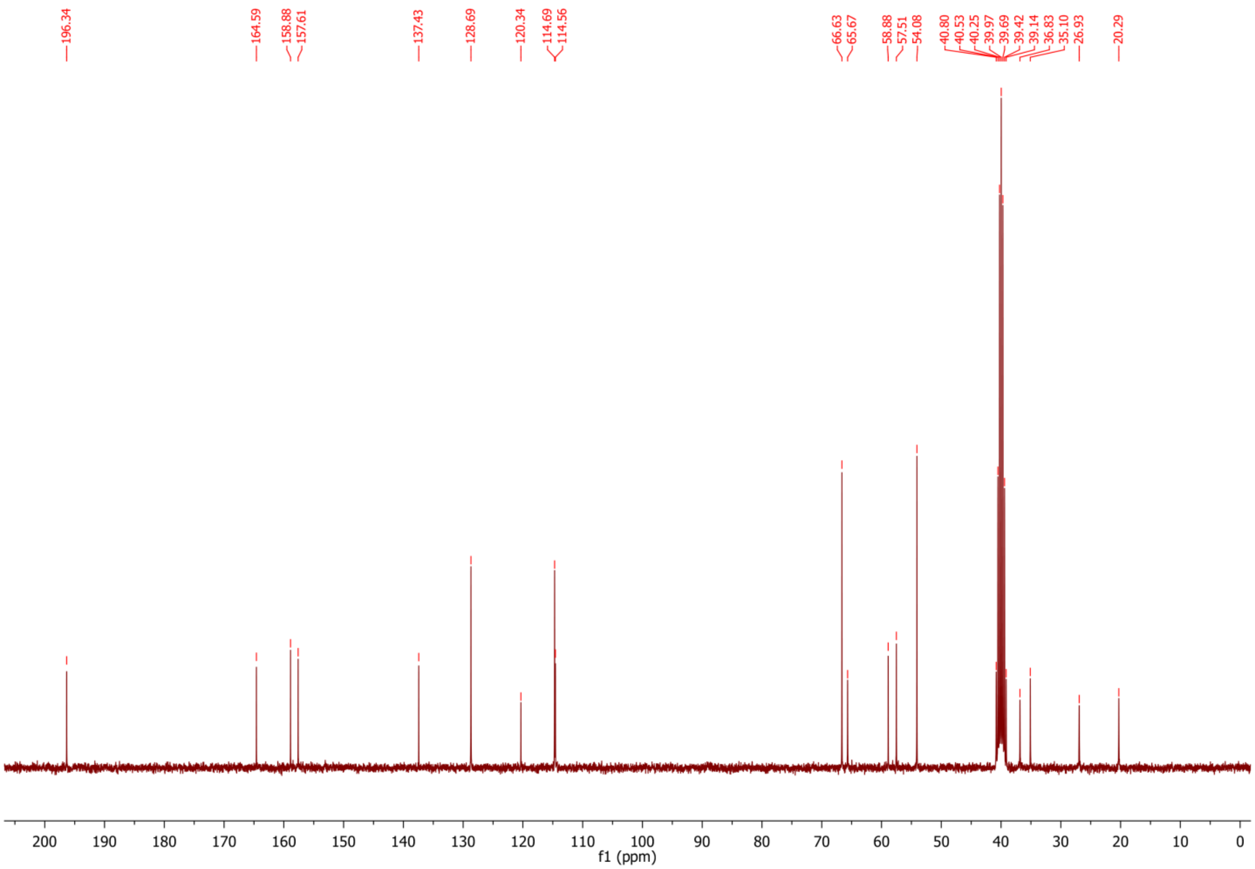

Fig. S6. ^{13}CNMR spectrum of product **4e**

Supporting information

2-Amino-4-{3-[(4-chlorobenzyl)oxy]phenyl}-5-oxo-5,6,7,8-tetrahydro-4H-chromene-3-carbonitrile (4f)

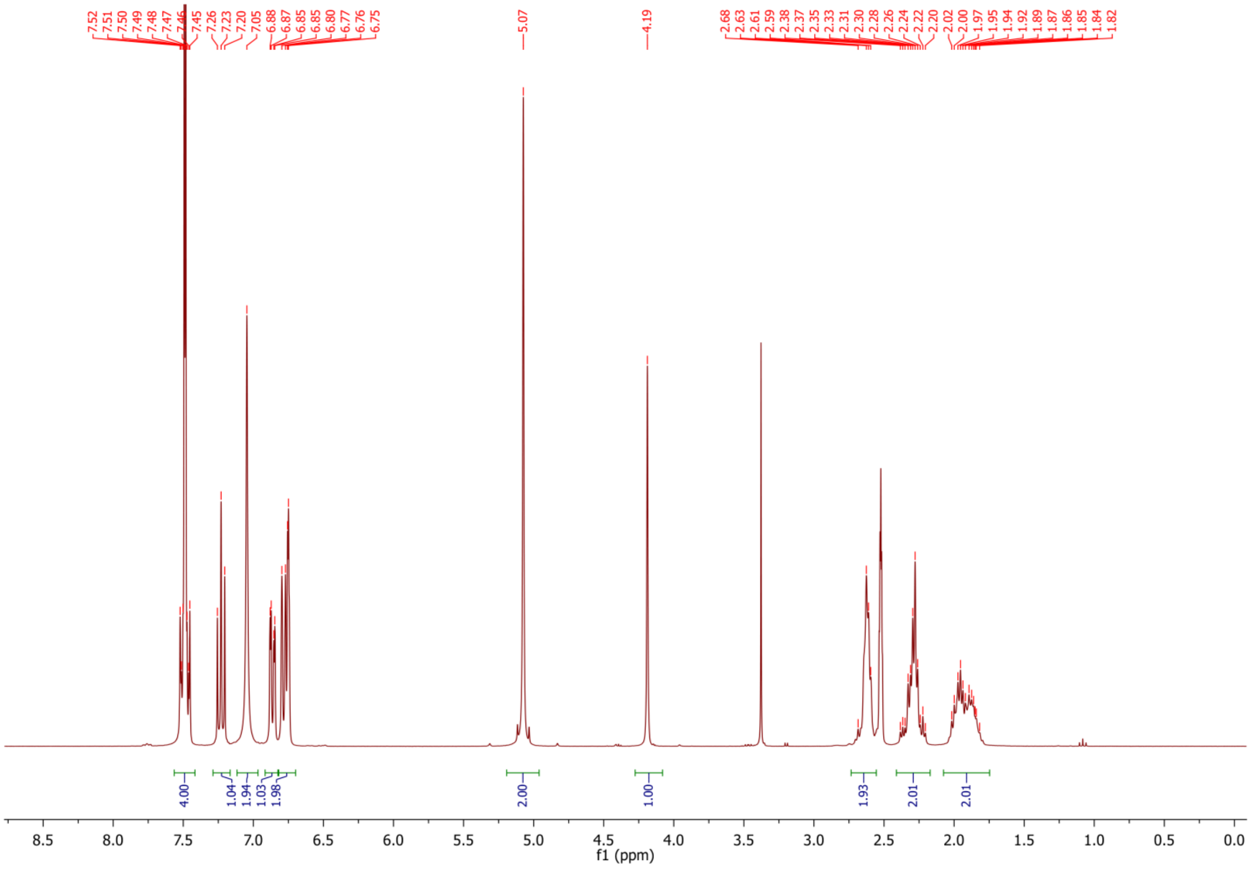

Fig. S7. ^{1}H NMR spectrum of product **4f**

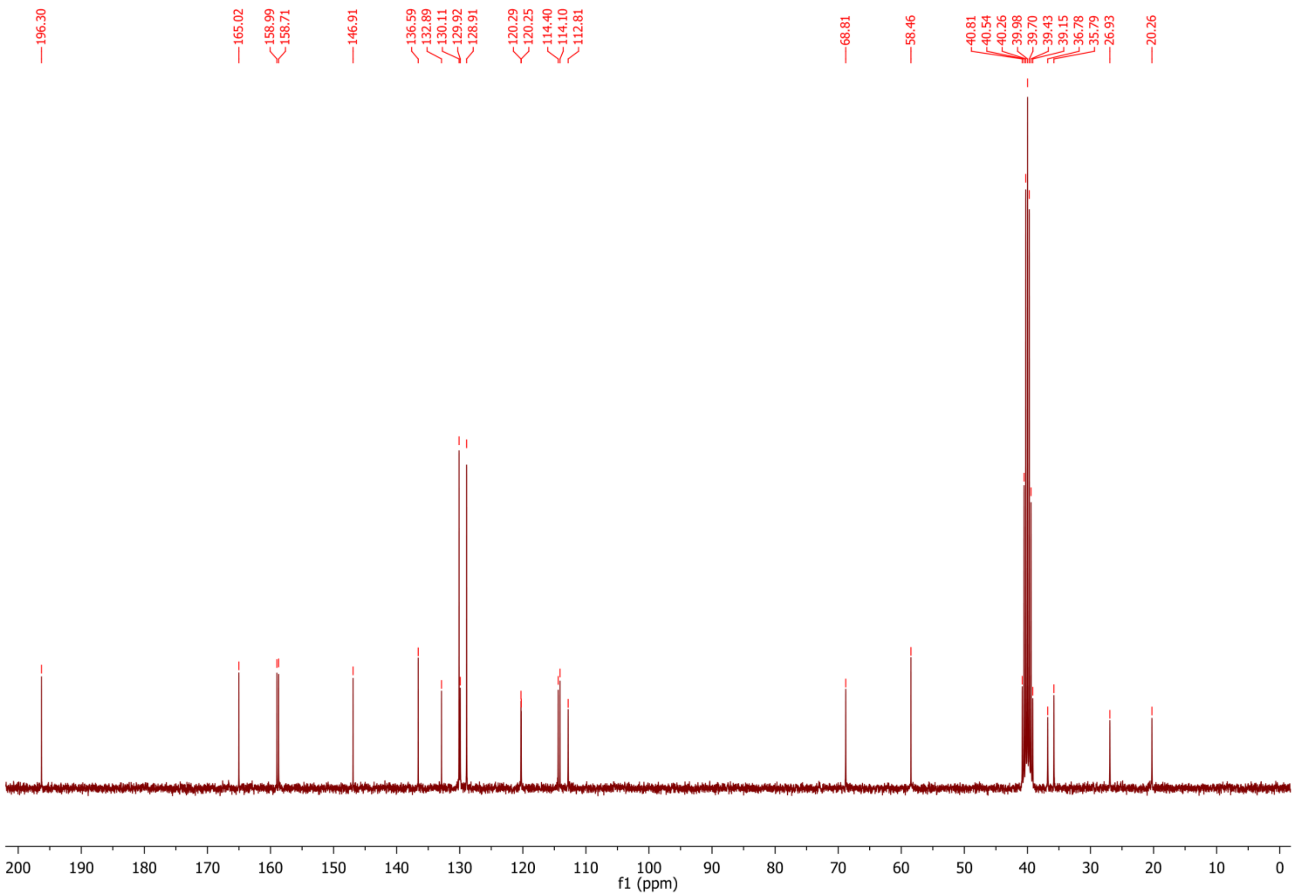

Fig. S8. ^{13}CNMR spectrum of product **4f**

2-Amino-4-{3-[(4-bromobenzyl)oxy]phenyl}-5-oxo-5,6,7,8-tetrahydro-4Hchromene-3-carbonitrile (4g)

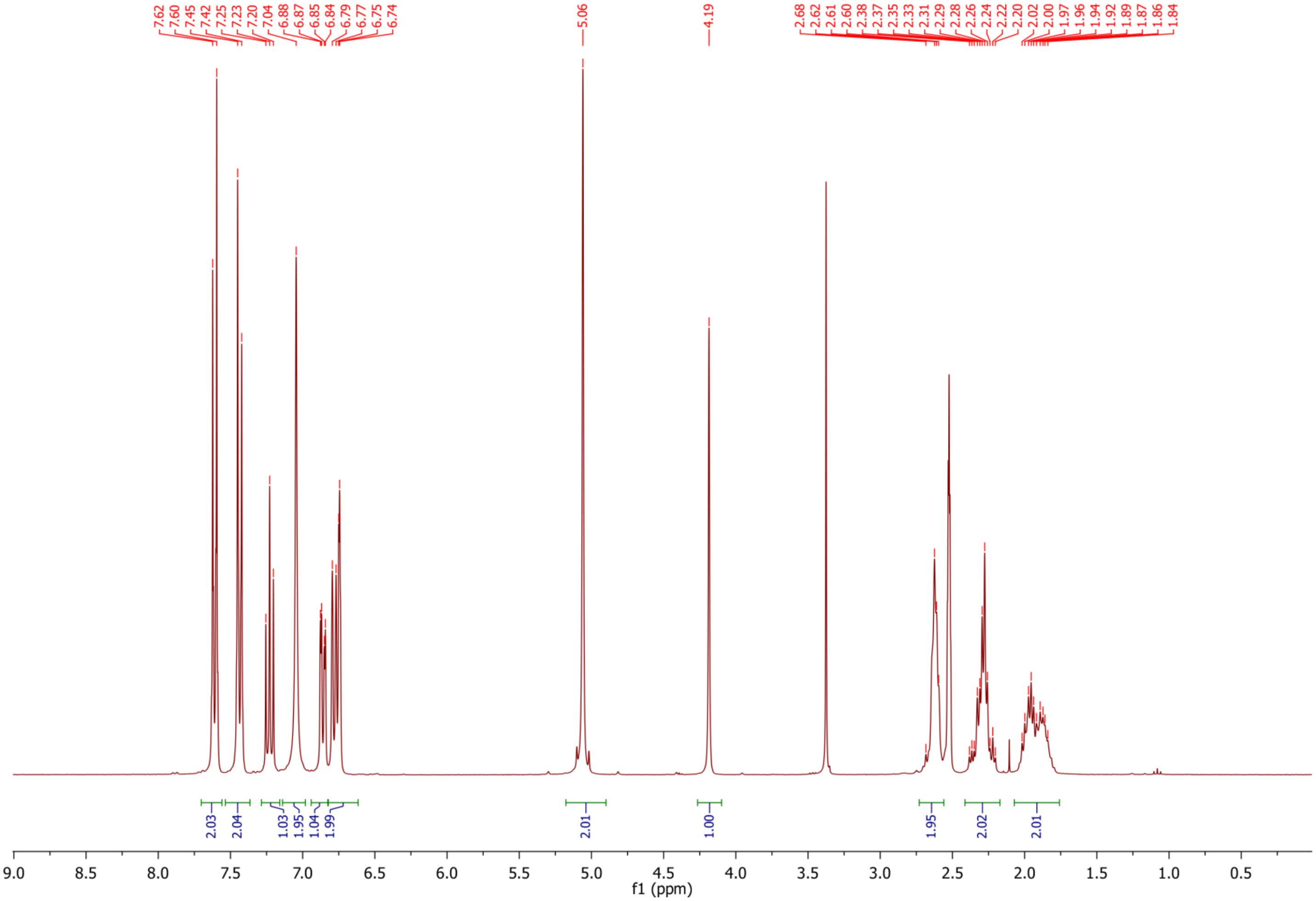

Fig. S9. ^{1}H NMR spectrum of product **4g**

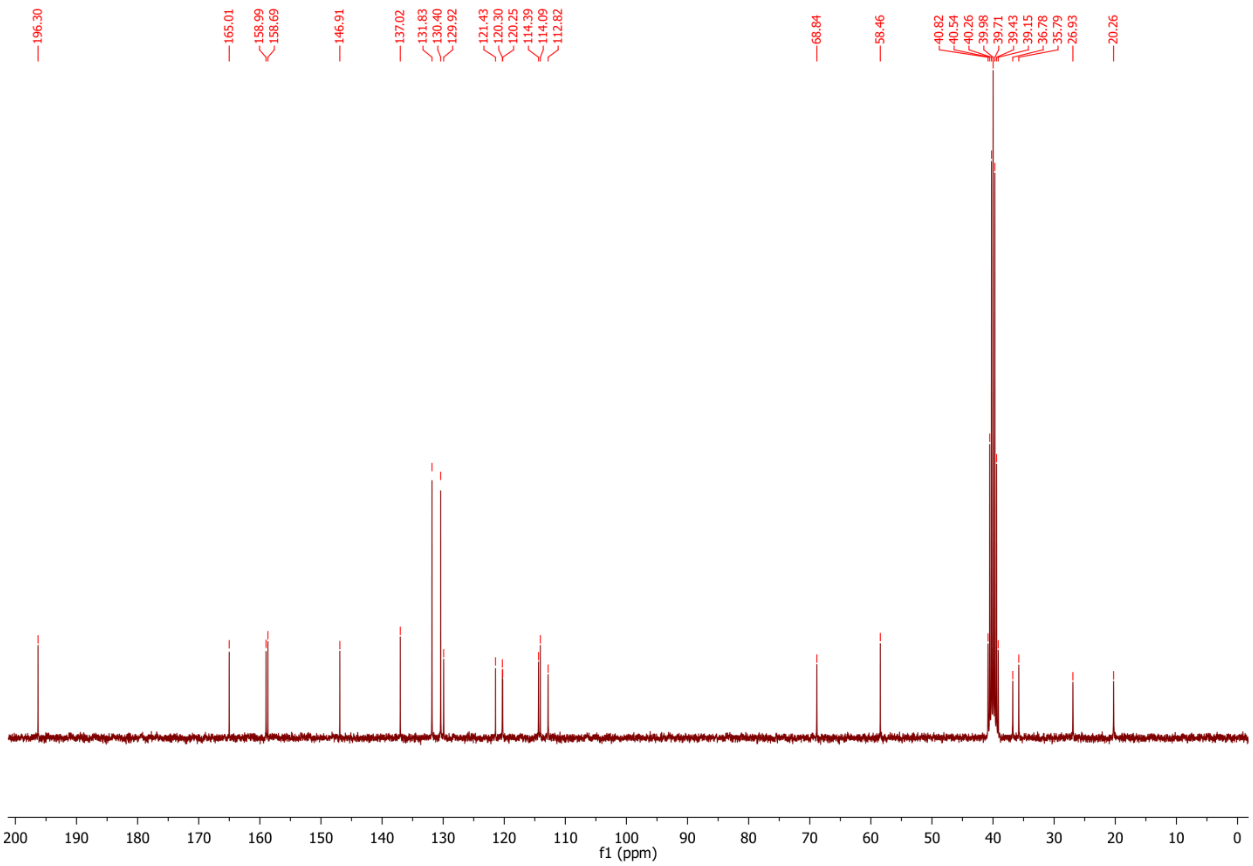

Fig. S10. ^{13}CNMR spectrum of product **4g**

Supporting information

2-Amino-4-{2-[(4-chlorobenzyl)oxy]phenyl}-5-oxo-5,6,7,8-tetrahydro-4H-chromene-3-carbonitrile (4h)

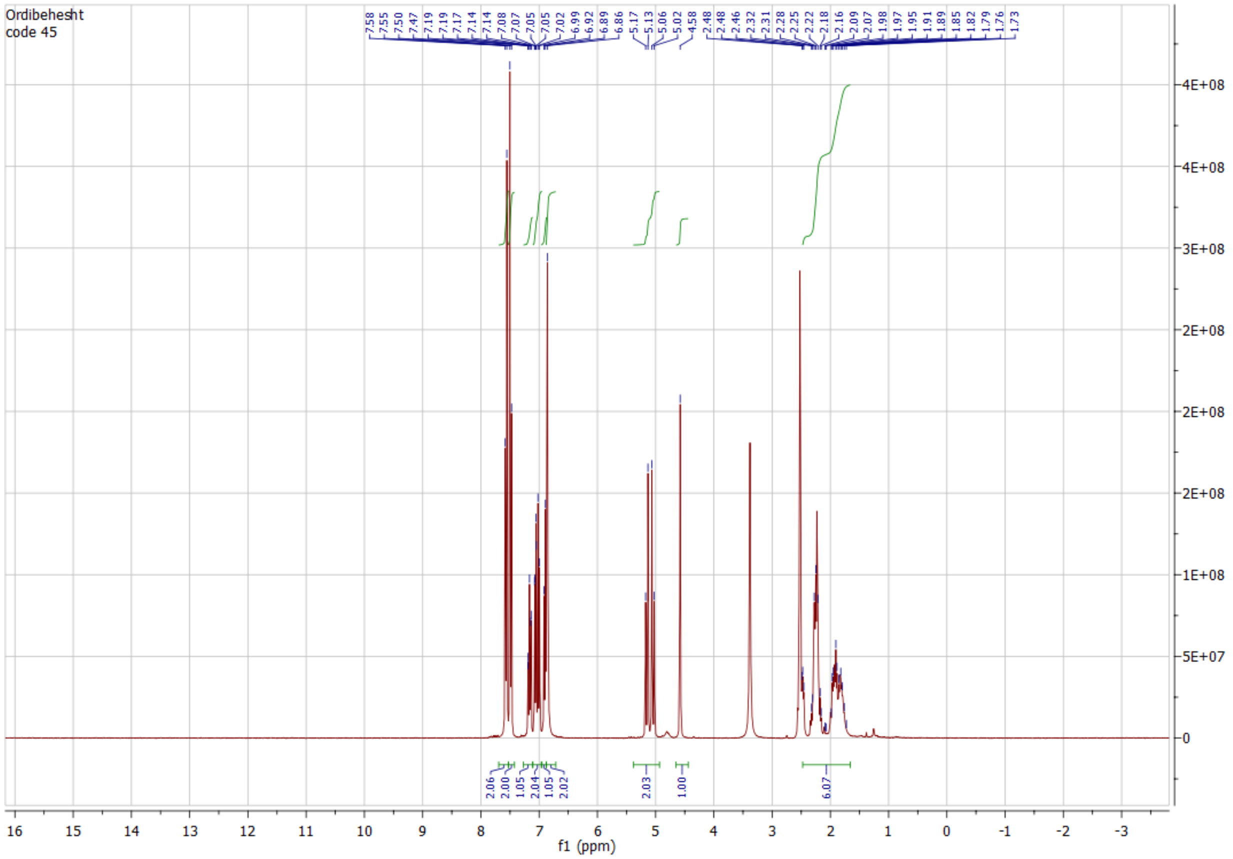

Fig. S11. ^{1}H NMR spectrum of product **4h**

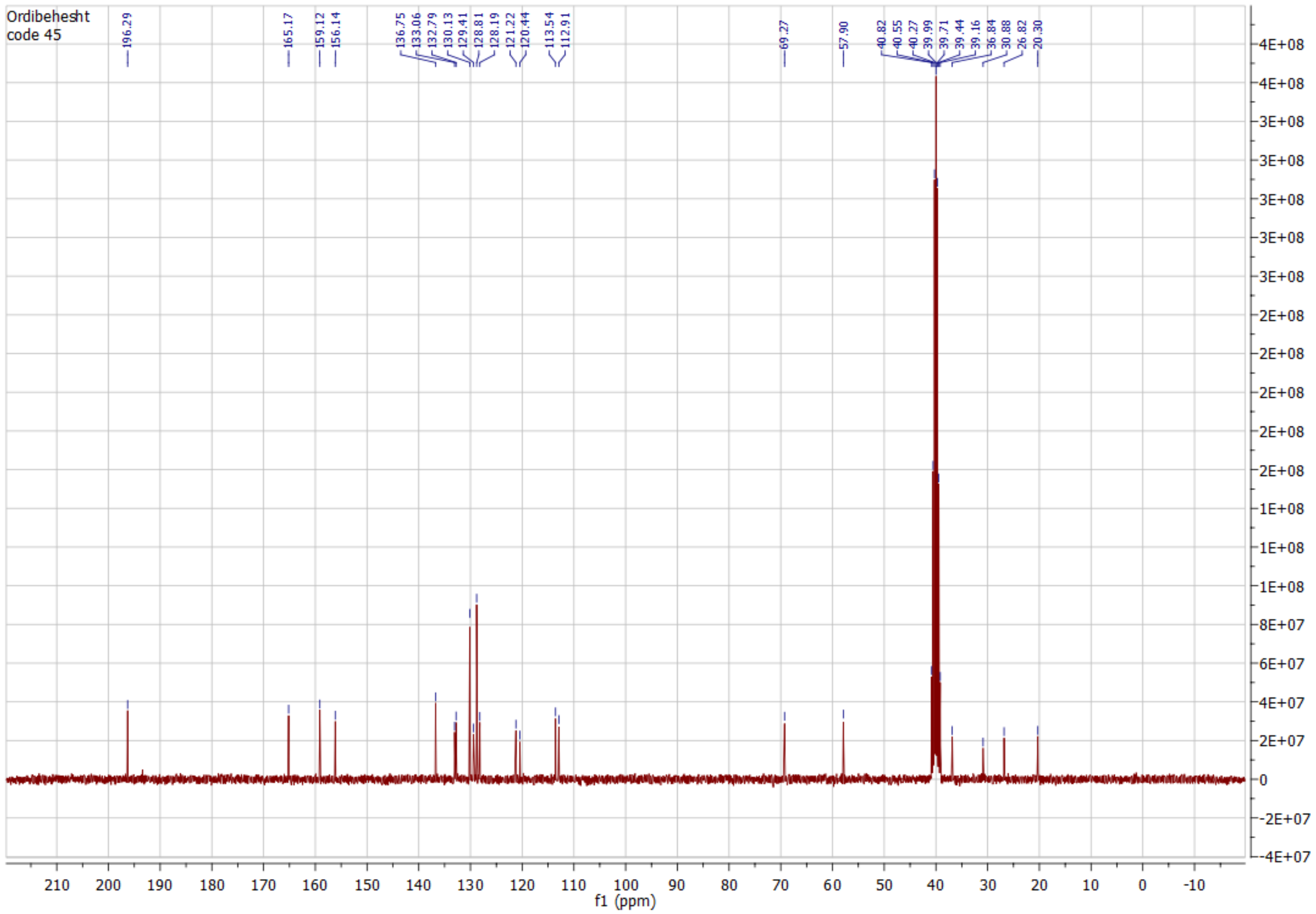

Fig. S12. ^{13}CNMR spectrum of product **4h**

Supporting information

2-Amino-4-{2-[(3-chlorobenzyl)oxy]phenyl}-5-oxo-5,6,7,8-tetrahydro-4H-chromene-3-carbonitrile(4i)

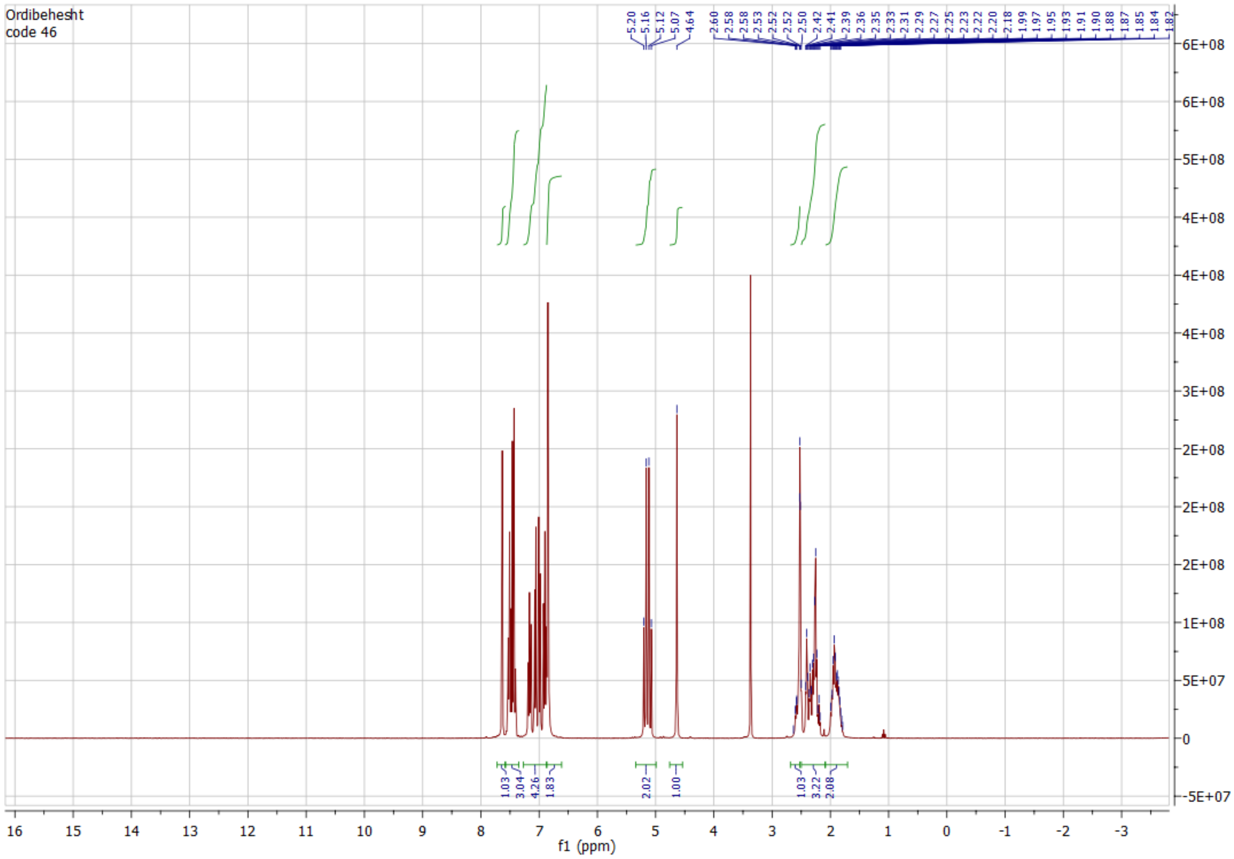

Fig. S13. ^{1}H NMR spectrum of product **4i**

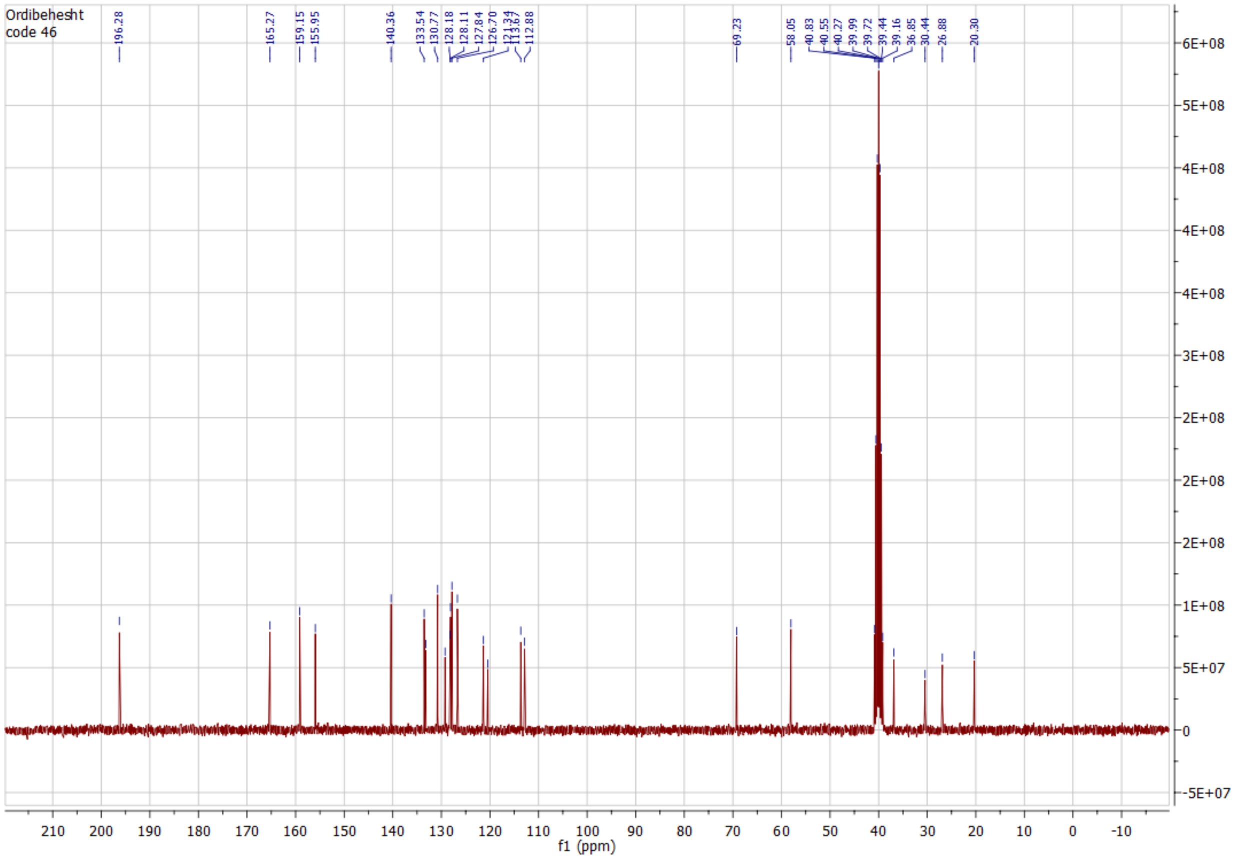

Fig. S14. ^{13}CNMR spectrum of product **4i**

2-Amino-4-{2-[(4-methoxybenzyl)oxy]phenyl}-5-oxo-5,6,7,8-tetrahydro-4H-chromene-3-carbonitrile(4j)

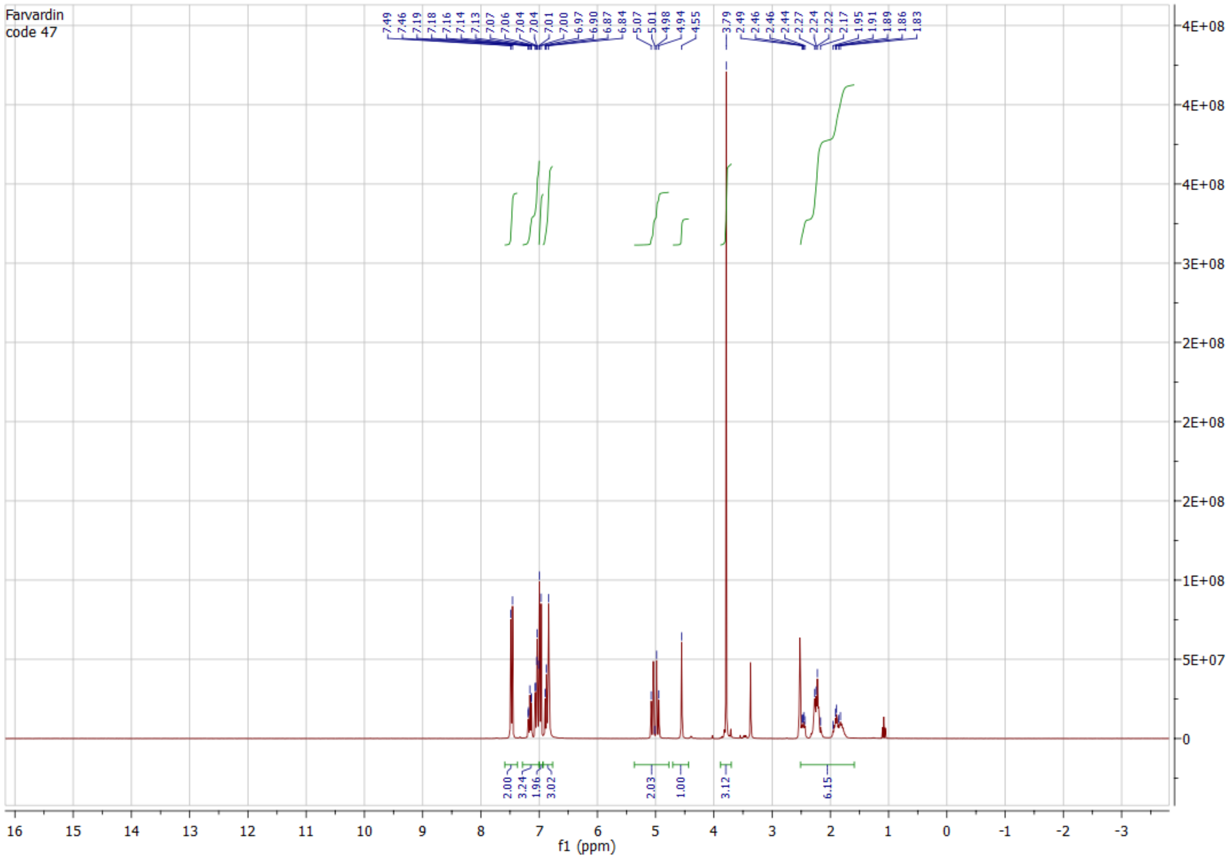

Fig. S15. ^{1}H NMR spectrum of product **4j**

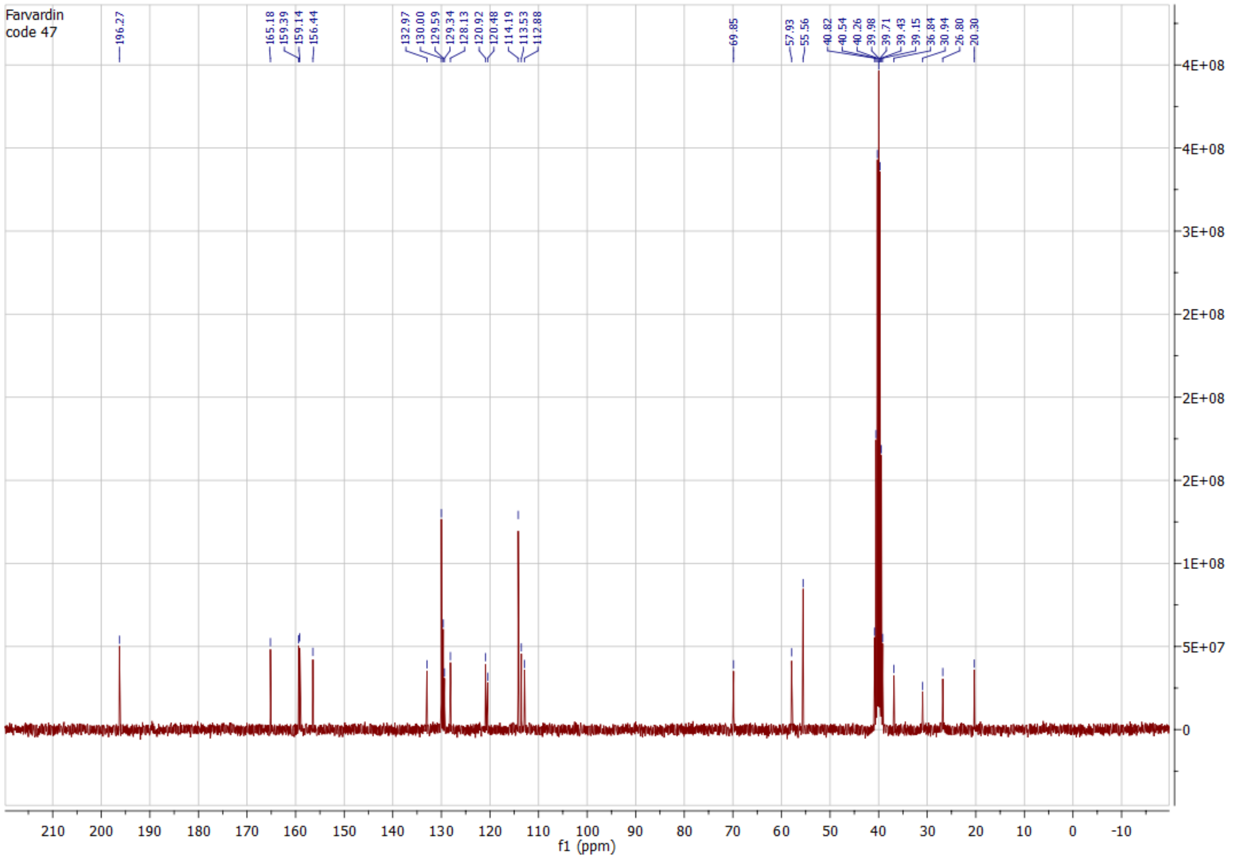

Fig. S16. ^{13}CNMR spectrum of product **4j**

2-Amino-4-{4-[(4-fluorobenzyl)oxy]-3-methoxyphenyl}-5-oxo-5,6,7,8-tetrahydro-4H-chromene-3-carbonitrile (4k)

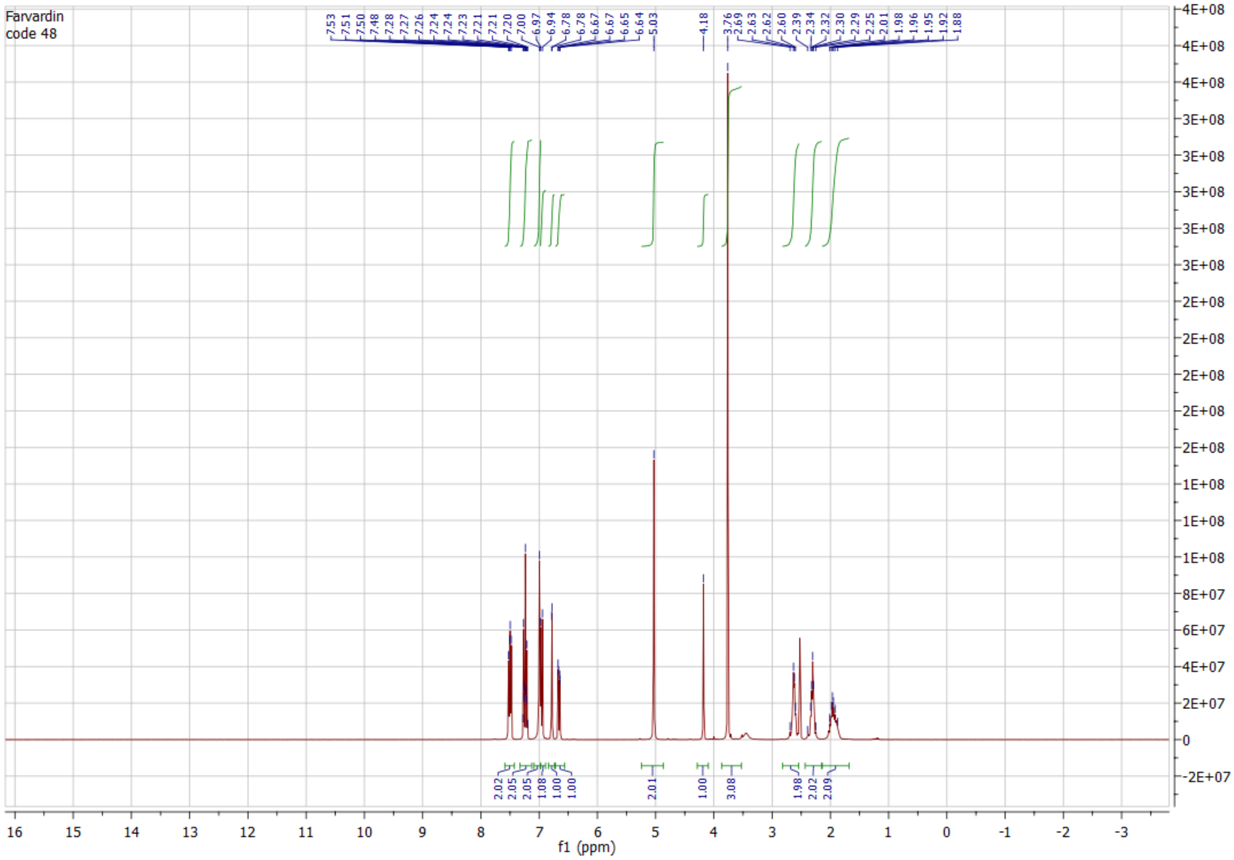

Fig. S17. ^{1}H NMR spectrum of product **4k**

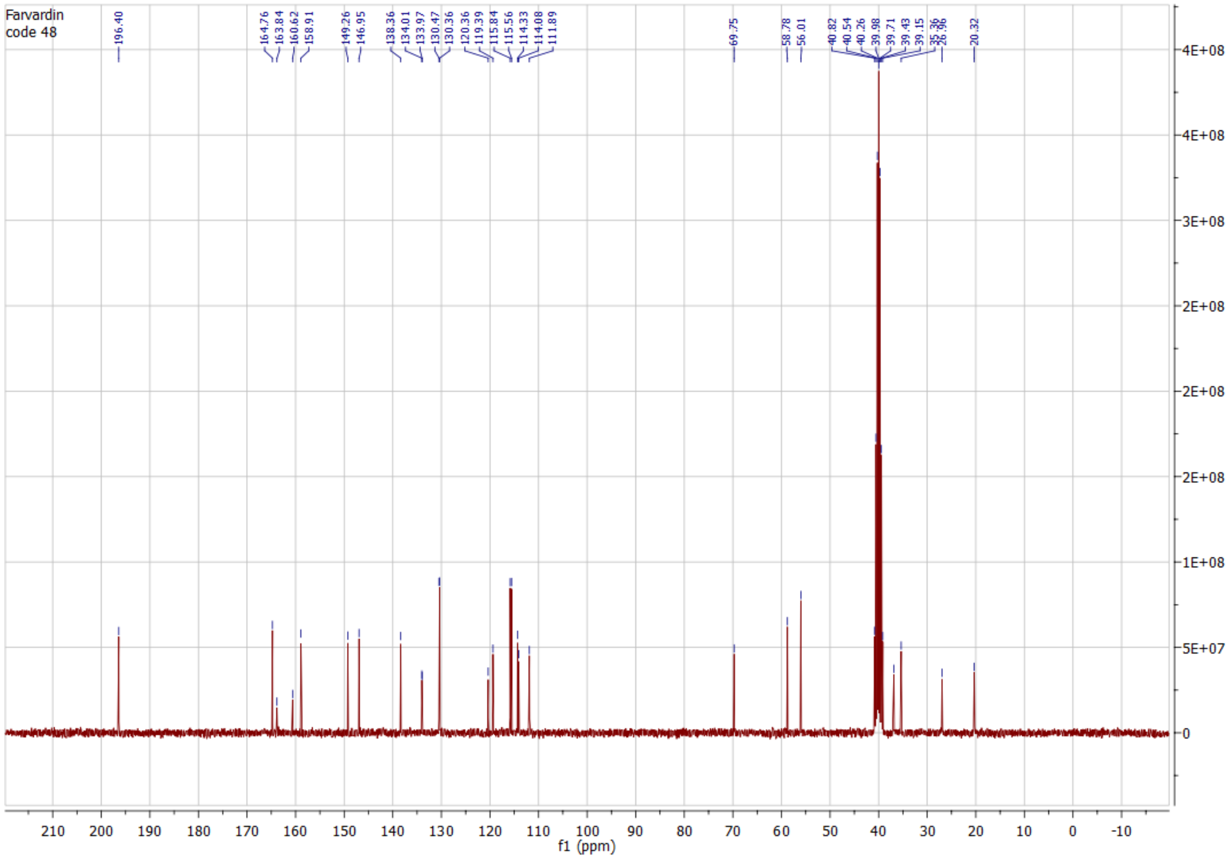

Fig. S18. ^{13}CNMR spectrum of product **4k**

2-Amino-4-{4-[(3-fluorobenzyl)oxy]-3-methoxyphenyl}-5-oxo-5,6,7,8-tetrahydro-4H-chromene-3-carbonitrile (4l)

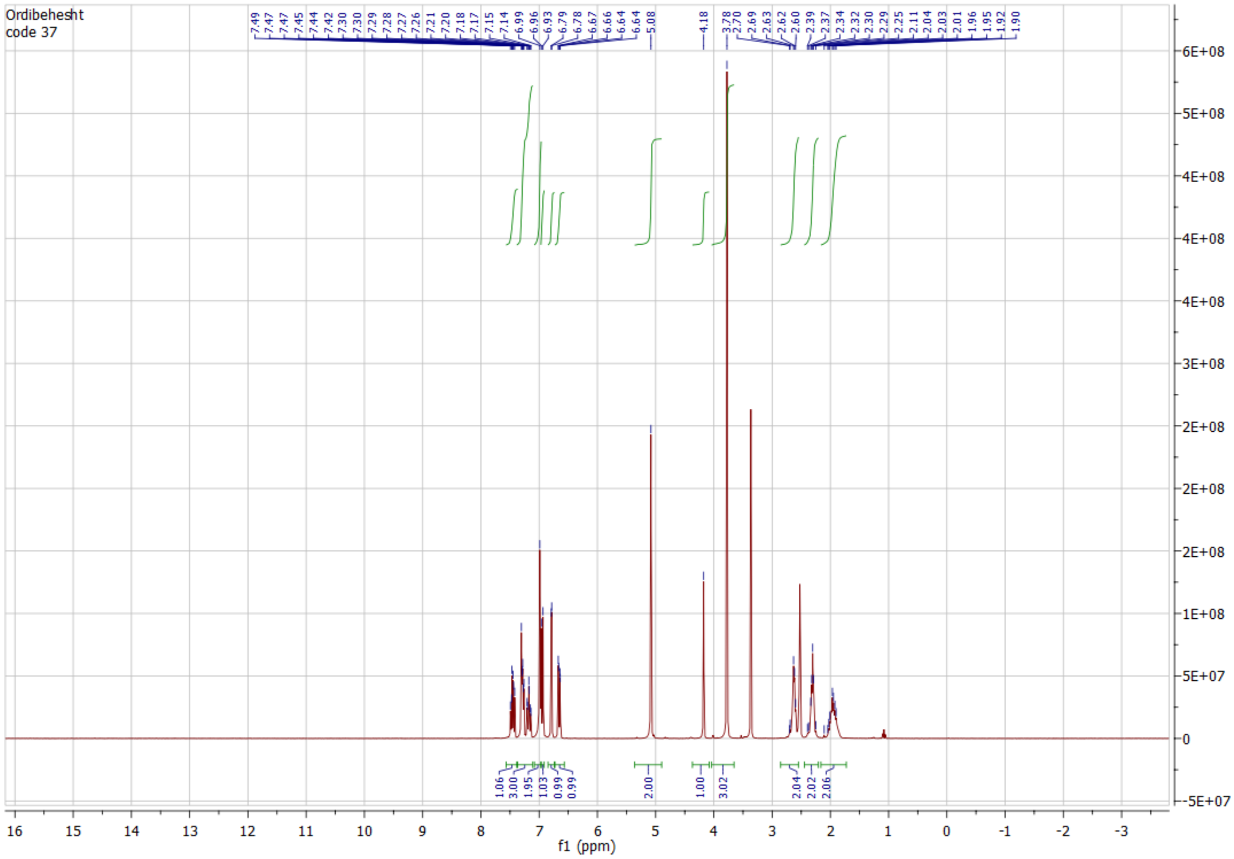

Fig. S19. ^{1}H NMR spectrum of product **4l**

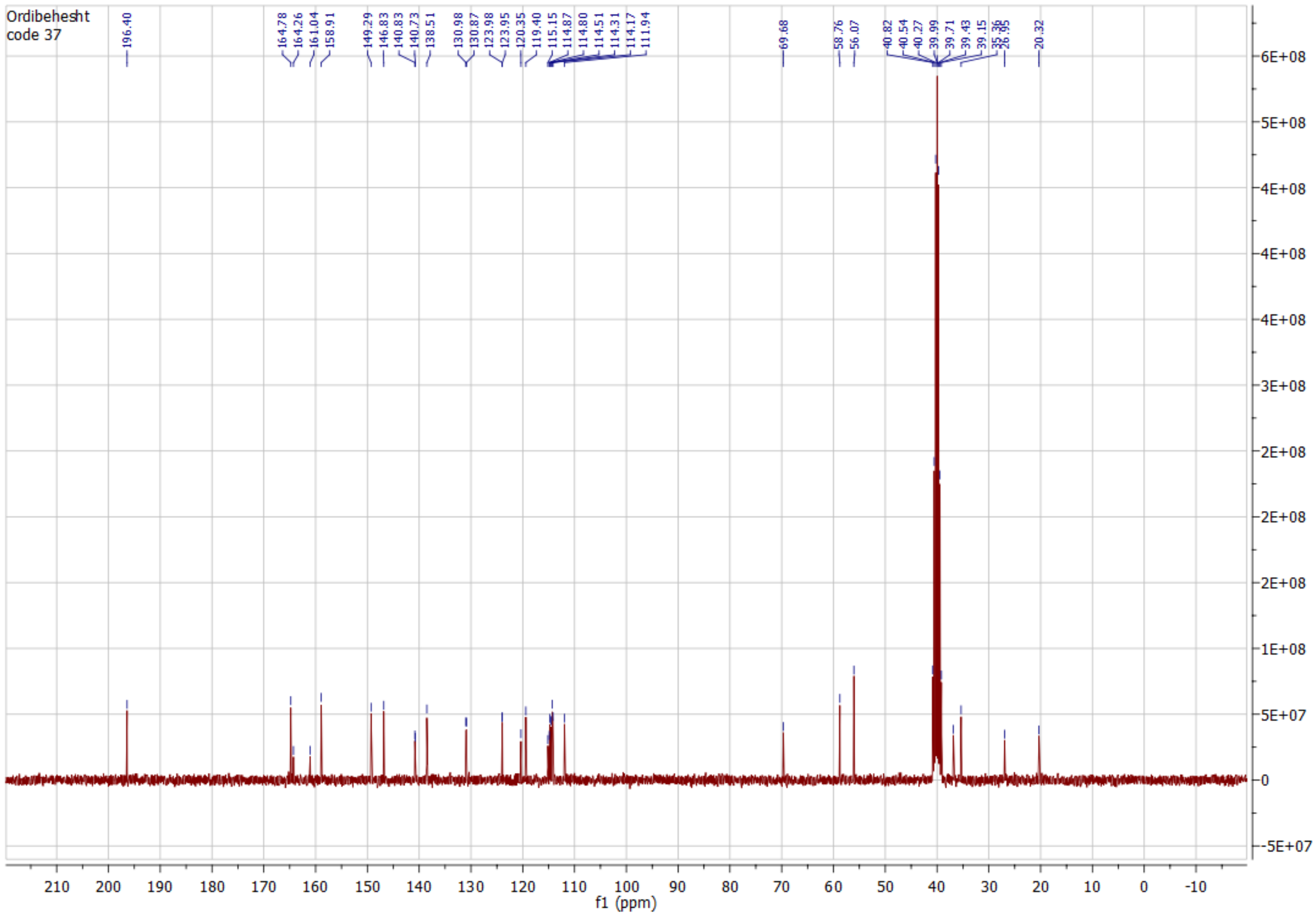

Fig. S20. ^{13}CNMR spectrum of product **4l**

A-amino-4-{4-[(3-chlorobenzyl)oxy]-3-methoxyphenyl}-5-oxo-5,6,7,8-tetrahydro-4H-chromene-3-carbonitrile (4m).

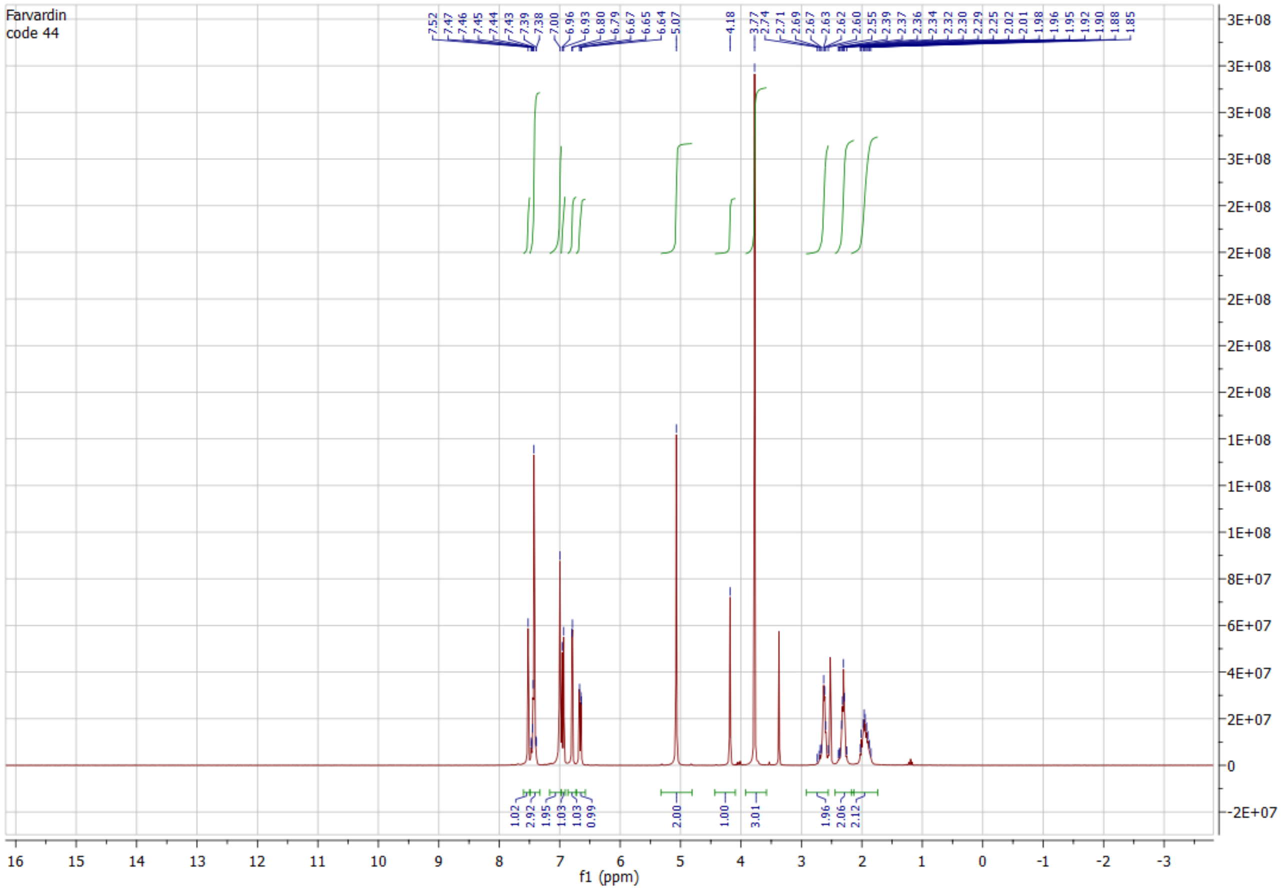

Fig. S21. ^{1}H NMR spectrum of product **4m**

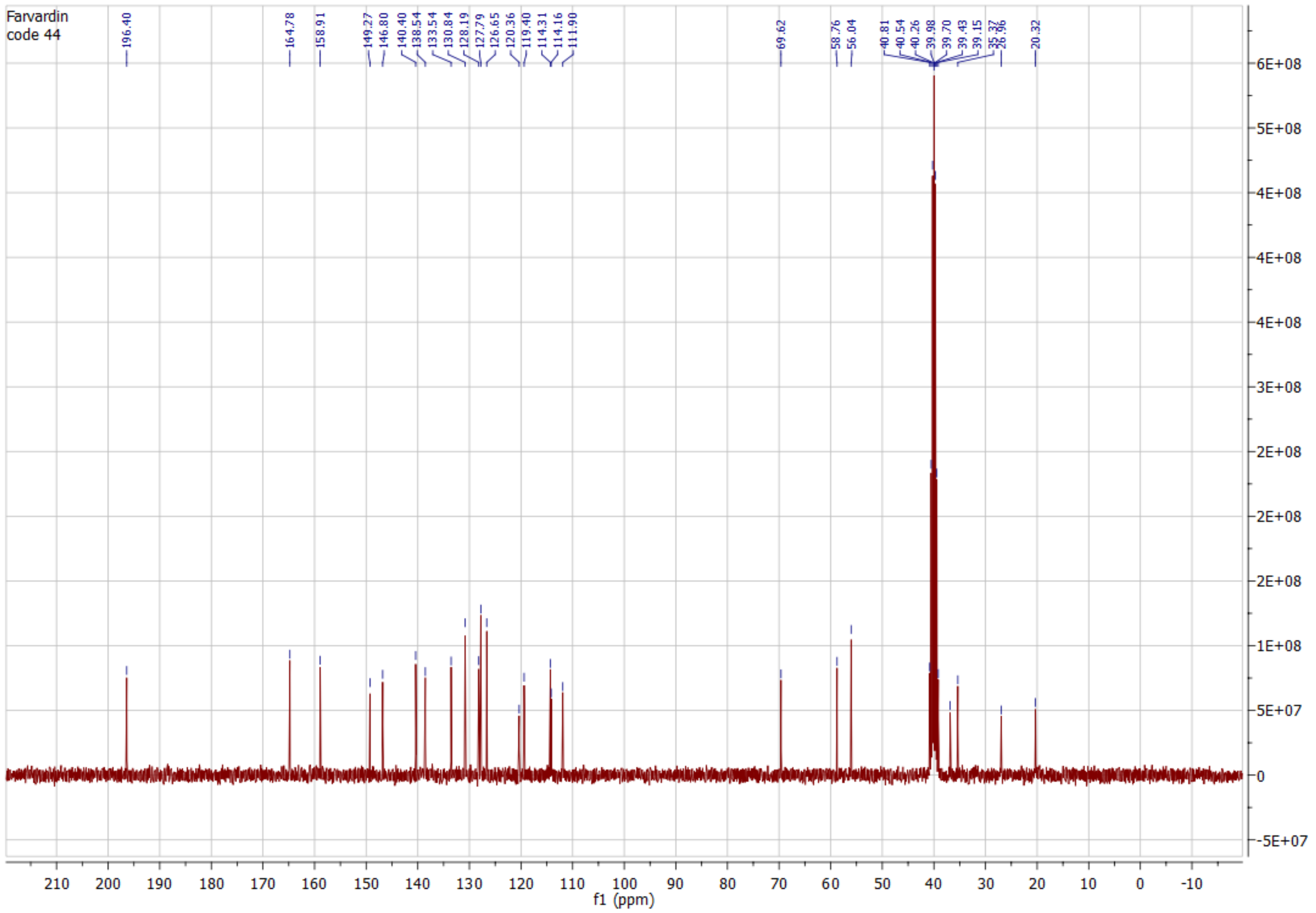

Fig. S22. ^{13}CNMR spectrum of product **4m**

Supporting information

Table S1. IC_{50} graph of potent compounds

<table>
<tr><td colspan="2">4b</td></tr>
<tr><td>
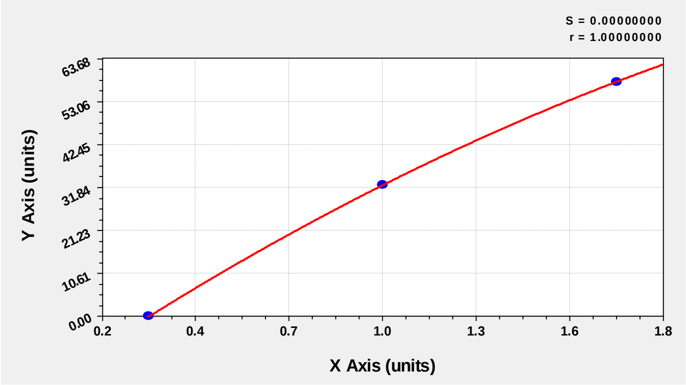

IC_{50}= 28.84 µM
</td><td>
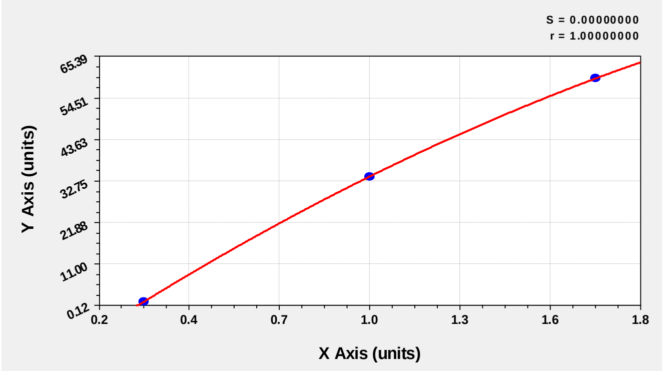

IC_{50}= 26.00 µM
</td></tr>
<tr><td colspan="2">4c</td></tr>
<tr><td>
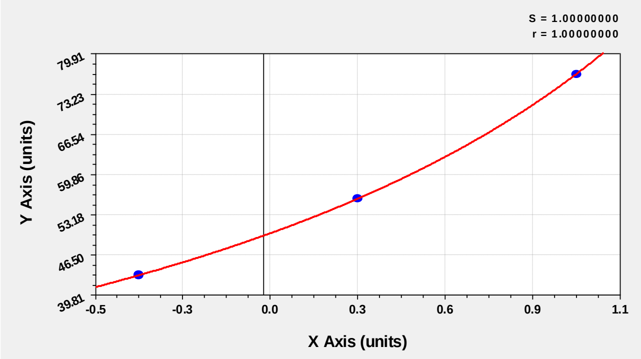

IC_{50}= 1.07 µM
</td><td>
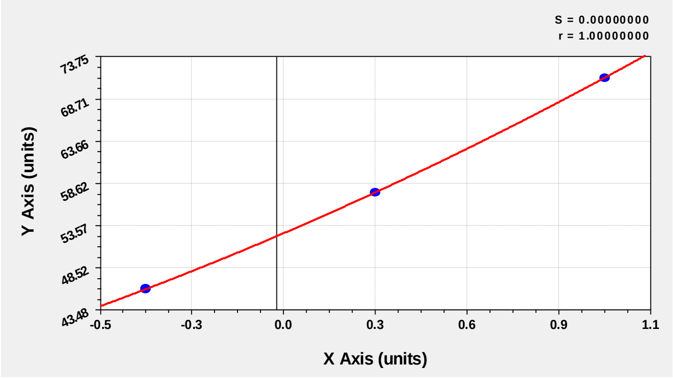

IC_{50}= 0.72 µM
</td></tr>
<tr><td colspan="2">4d</td></tr>
<tr><td>
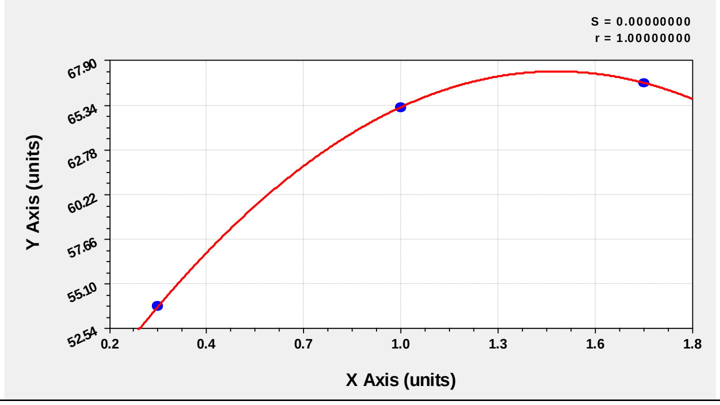

</td><td>
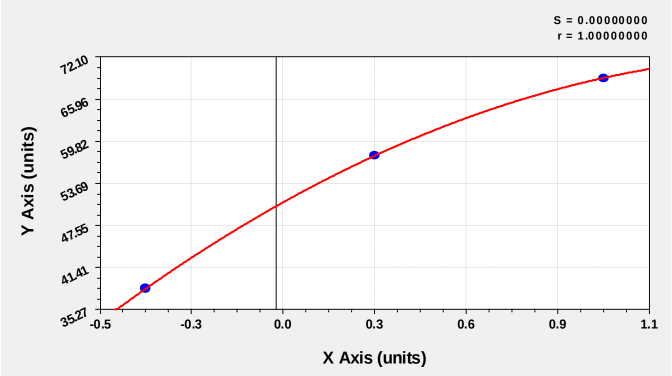

</td></tr>
<tr><td>1.41 µM</td><td>0.97 µM</td></tr>
</table>

Supporting information

4e

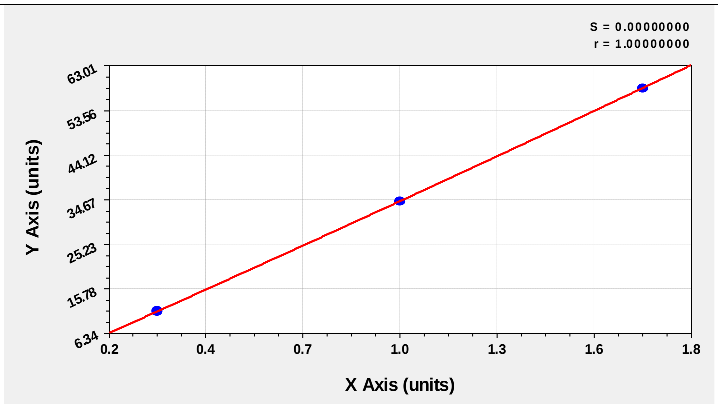

IC_{50} = 28.71 µM

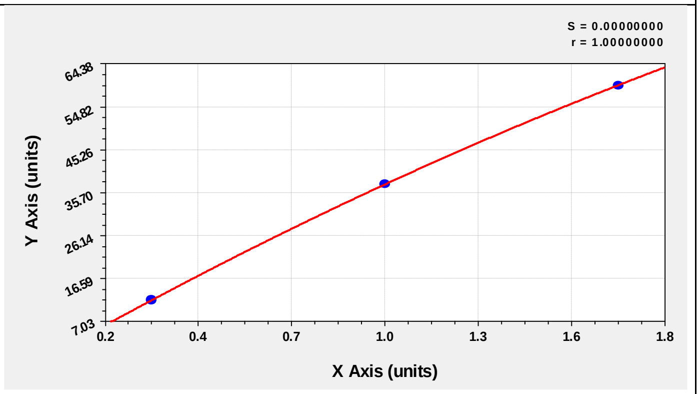

IC_{50} =23.99 µM

4f

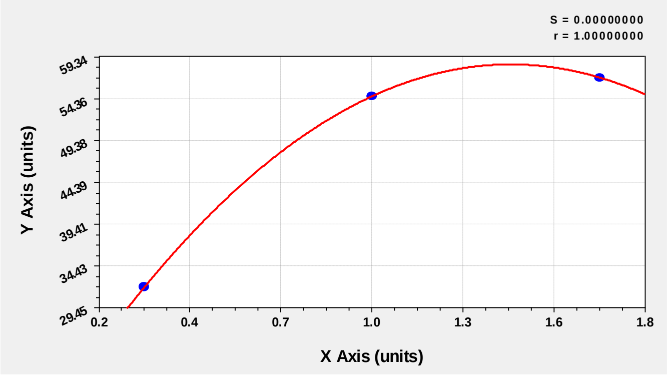

IC_{50}= 6.18 µM

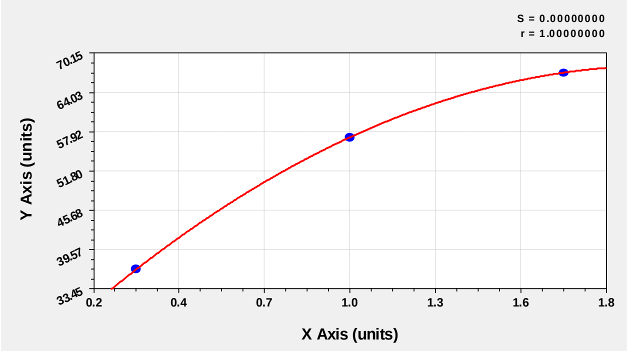

IC_{50}= 5.21 µM

4g

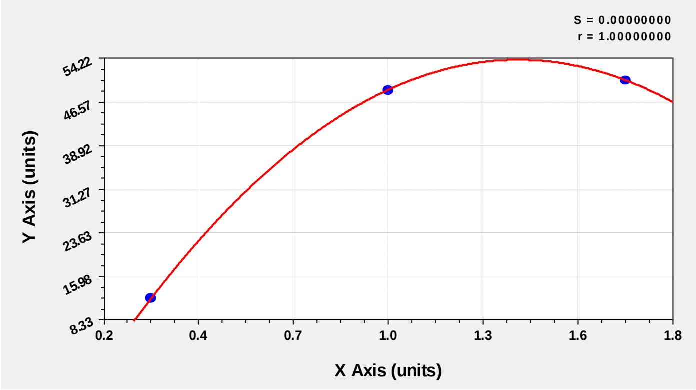

IC_{50}= 11.22 µM

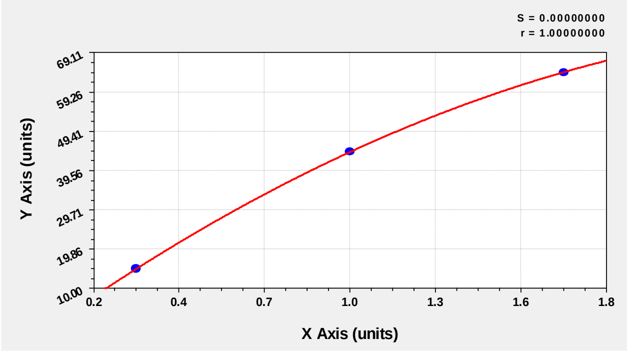

IC_{50}= 14.89 µM

Supporting information

4l

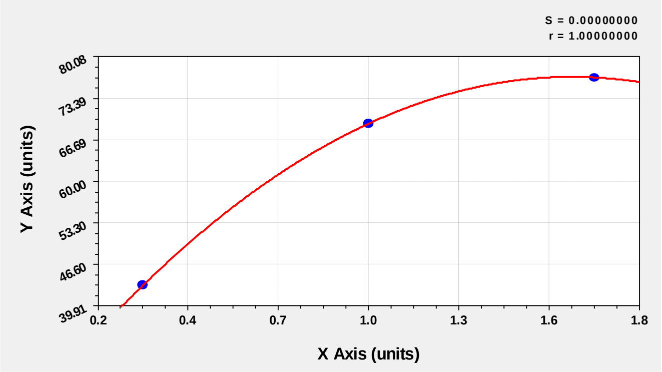

IC_{50}= 2.77 μM

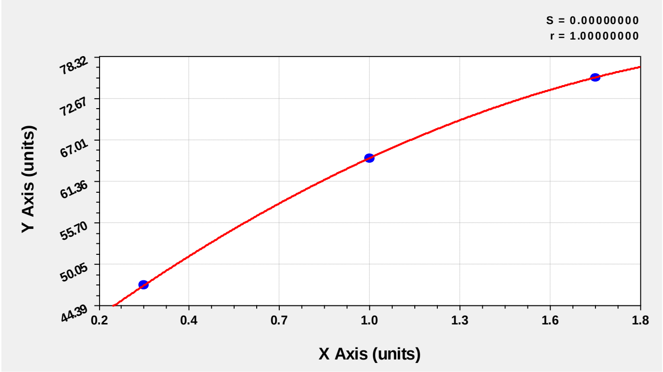

IC_{50}= 2.49 μM

4m

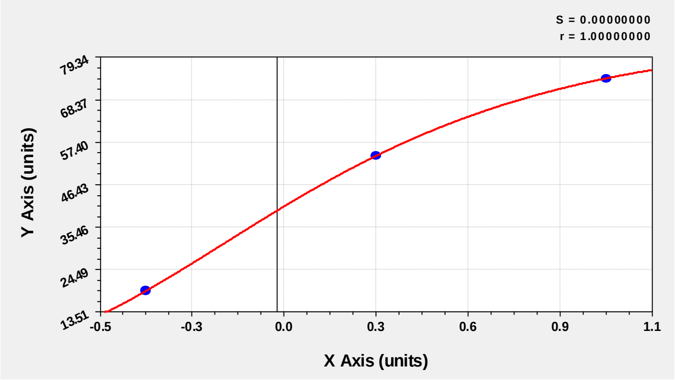

IC_{50}= 1.65 μM

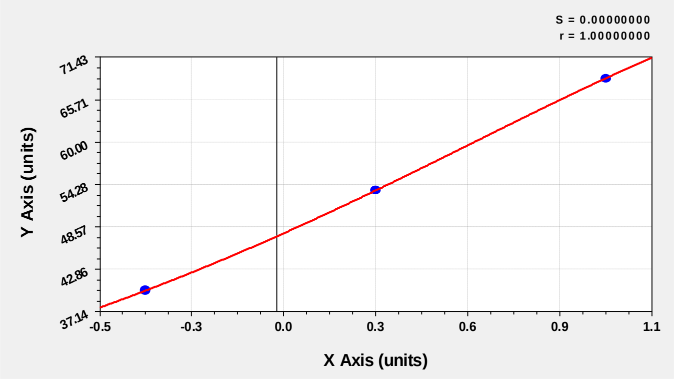

IC_{50}= 1.36 μM
